# Antimicrobial Diterpenes from Rough Goldenrod (*Solidago rugosa* Mill.)

**DOI:** 10.3390/molecules28093790

**Published:** 2023-04-28

**Authors:** Márton Baglyas, Péter G. Ott, Ildikó Schwarczinger, Judit Kolozsváriné Nagy, András Darcsi, József Bakonyi, Ágnes M. Móricz

**Affiliations:** 1 Plant Protection Institute, Centre for Agricultural Research, ELKH, Herman O. Str. 15, 1022 Budapest, Hungary; baglyas.marton@atk.hu (M.B.); ott.peter@atk.hu (P.G.O.); schwarczinger.ildiko@atk.hu (I.S.); nagy.judit@atk.hu (J.K.N.); bakonyi.jozsef@atk.hu (J.B.); 2 Doctoral School of Pharmaceutical Sciences, Semmelweis University, Hőgyes E. Str. 7-9, 1092 Budapest, Hungary; 3 Pharmaceutical Chemistry and Technology Department, National Institute of Pharmacy and Nutrition, Szabolcs Str. 33, 1135 Budapest, Hungary; darcsi.andrew@gmail.com

**Keywords:** high-performance thin-layer chromatography–effect-directed analysis, bioassay-guided isolation, antibacterial effect, antifungal effect, (–)-hardwickiic acid, (–)-abietic acid

## Abstract

*Solidago rugosa* is one of the goldenrod species native to North America but has sporadically naturalized as an alien plant in Europe. The investigation of the root and leaf ethanol extracts of the plant using a bioassay-guided process with an anti-*Bacillus* assay resulted in the isolation of two antimicrobial components. Structure elucidation was performed based on high-resolution tandem mass spectrometric and one- and two-dimensional NMR spectroscopic analyses that revealed (–)-hardwickiic acid (Compound **1**) and (–)-abietic acid (Compound **2**). The isolates were evaluated for their antimicrobial properties against several plant pathogenic bacterial and fungal strains. Both compounds demonstrated an antibacterial effect, especially against Gram-positive bacterial strains (*Bacillus spizizenii*, *Clavibacter michiganensis* subsp. *michiganensis*, and *Curtobacterium flaccumfaciens* pv. *flaccumfaciens*) with half maximal inhibitory concentration (IC_50_) between 1 and 5.1 µg/mL (5–20 times higher than that of the positive control gentamicin). In the used concentrations, minimal bactericidal concentration (MBC) was reached only against the non-pathogen *B. spizizenii*. Besides their activity against *Fusarium avenaceum*, the highest antifungal activity was observed for Compound **1** against *Bipolaris sorokiniana* with an IC_50_ of 3.8 µg/mL.

## 1. Introduction

**Figure 1:**
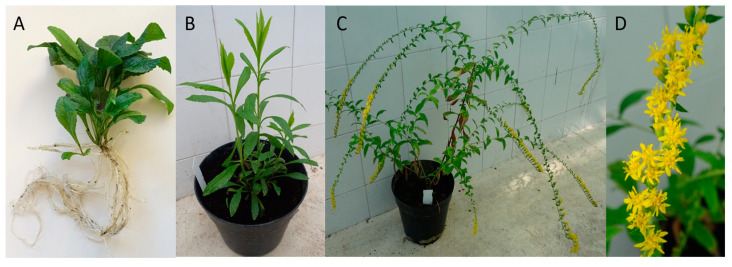
Young shoots with root (**A**), young (**B**), and flowering (**C**) plants and cascading flowerhead (**D**) of *Solidago rugosa*.

Goldenrod plants belong to the genus *Solidago* which includes over 120 members, with the majority being native to North America. These herbaceous perennial plants with yellow flowers often grow up to 2 m in height. *Solidago virgaurea* L. is the only native goldenrod in Europe, but several North American goldenrods were introduced to Europe as ornamentals, and at least four of these species have become naturalized. *S. gigantea* Aiton and *S. canadensis* L. became particularly successful invaders, while *S. graminifolia* (L.) Elliot and *S. rugosa* Mill. occur only sporadically [1,2,3]. *S. rugosa* (rough or wrinkle-leaved goldenrod) is considered a naturalized alien already in Switzerland, Portugal, Norway, and Great Britain, probably present in France and Belgium [3,4,5] and its geographic expansion is still in progress. The plant has hairy stems lined with thick, firm, and rough-textured leaves, and at the tips, yellow flowerheads cascade (Figure 1). Only limited information is available regarding the bioactivity of the rough goldenrod’s chemical constituents. It was observed that, compared to other *Solidago* species, *S. rugosa* was more resistant against the rust fungus *Coleosporium asterum*, with a lower incidence of bright orange rust pustules on the leaves [6]. Furthermore, extracts of rough goldenrod tissues inhibited *Mycobacterium tuberculosis* in the decreasing order of root, flower, and stem [7]. In both root and leaf, the presence of terpenes was reported, including essential oil components [8] and diterpenes [9,10].

Despite the occasional occurrence of hypersensitivity reactions or gastrointestinal disorders, the aerial parts of several other *Solidago* species are traditionally used for the treatment of, e.g., minor urinary complaints and diabetes [11,12]. Goldenrod extracts showed a wide range of pharmacological effects, including antimicrobial, insecticidal, anti-obesity, antimutagenic, anti-inflammatory, and cholinesterase inhibitory activity [11,13,14,15,16] that can be attributed to the secondary metabolites of the plants, such as phenolic acids, flavonoids, essential oils, polyacetylenes, diterpenes, triterpene saponins, and tannins [11,13,17,18,19,20].

Phytopathogenic fungi and bacteria have shown a tendency to develop resistance to pesticides due to adaptive mutations as a consequence of the extensive use of plant protection agents [21,22]. The loss of efficacy of these agrochemicals poses a significant threat to crop production, resulting in poor-quality products, lower yields, and an increased risk of plant diseases [23]. Furthermore, it has a negative impact on human nutrition and also raises environmental and ecological concerns. Several modes of action of antimicrobial molecules are known, although microbes are increasingly evading these mechanisms [22]. Therefore, to find sustainable solutions for managing pesticide resistance is critical for maintaining crop productivity. There is an urgent demand for extending the chemical space by discovering new, effective, and environmentally friendly substances. Plants are considered an inexhaustible source of secondary metabolites possessing valuable, diverse structures and biological activity [24,25]. Natural products isolated from plants can serve as starting points for chemical modifications to improve their properties, acting as lead compounds during the development of small-molecule, potent antimicrobial biopesticides [26].

High-performance thin-layer chromatography coupled with effect-directed analysis (HPTLC–EDA) is a quick, straightforward, cost-efficient, and powerful hyphenated method for the non-targeted, high-throughput screening of plant extracts for bioactive compounds, avoiding the commonly used, suboptimal trial and error approach in the labor-intensive, expensive isolation procedure [13,27]. After the determination of the bioprofile of a sample by an HPTLC–direct bioautographic (DB) method [28], the detected inhibition zones can be characterized by various techniques, such as HPTLC–mass spectrometry (MS) [29]. Therefore, HPTLC hyphenations combined with preparative column chromatographic fractionations can contribute to the detection and subsequent separation, purification, and isolation of bioactive compounds from challenging matrices [30].

This study aimed to detect, isolate, and identify the antimicrobial root and leaf components of *S. rugosa* by the combination of a non-targeted, effect-directed screening and a highly targeted, bioassay-guided isolation involving high-performance thin-layer chromatography (HPTLC)–*Bacillus subtilis* assay, preparative flash chromatography, HPLC–high-resolution tandem mass spectrometry (HRMS/MS), and nuclear magnetic resonance (NMR) spectroscopy. The bioactivity of the isolated compounds was characterized by the determination of the minimal bactericidal concentration (MBC), the minimal inhibitory concentration (MIC), and the half maximal inhibitory concentration (IC_50_) values mainly against plant pathogens.

## 2. Results and Discussion

**Figure 2:**
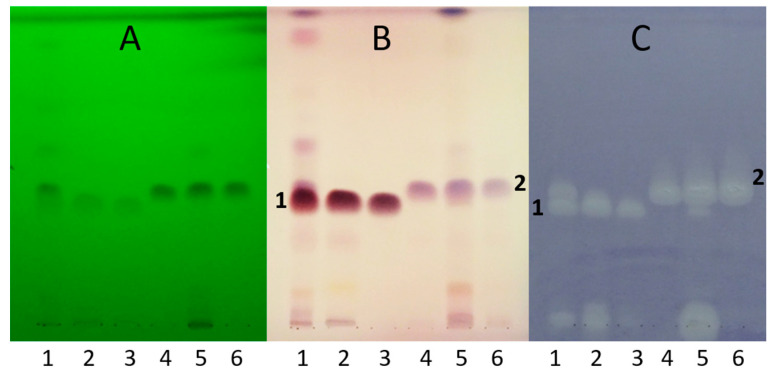
HPTLC chromatograms of *S. rugosa* root (1) and leaf (5) extracts, their main flash fractions (2 and 6, respectively), and Isolates **1** (3) and **2** (4), developed with *n*-hexane–isopropyl acetate 4:1 *v*/*v*, and detected at UV 254 nm (**A**), at white light illumination after derivatization with vanillin–sulfuric acid reagent (**B**), and bioautogram after *Bacillus subtilis* assay (**C**).

HPTLC separation using *n*-hexane–isopropyl acetate 4:1 *v*/*v* mobile phase and detection with vanillin–sulfuric acid reagent and *B. subtilis* bioassay revealed active zones in both root and leaf at *hR*_F_ 38 and 43, Compounds **1** and **2**, respectively (Figure 2). Both compounds were present in both tissues, but the root was richer in Compound **1**, while Compound **2** was more prevalent in the leaves. Their isolation was achieved by preparative flash chromatographic fractionation and purification applying consecutive, orthogonal separation steps, switching from normal-phase silica gel to C_18_ reversed-phase stationary phase and back to silica gel columns, yielding 25.5 mg of Compound **1** (white powder) and 9.4 mg of Compound **2** (pale yellow powder). Their structural characterization was performed based on mass spectrometric and NMR spectroscopic data.

**Figure 3:**
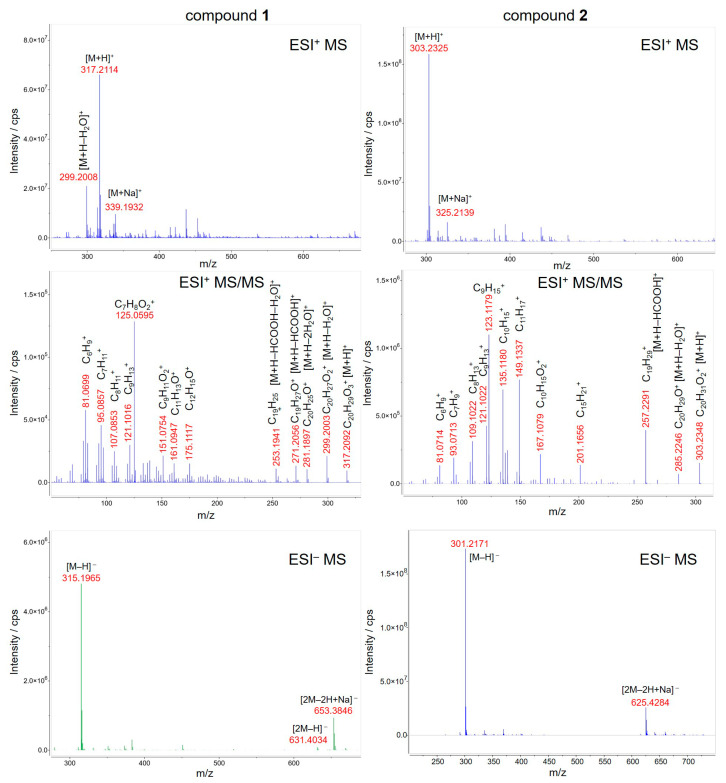
HPLC–ESI-qTOF-MS(/MS) spectra of Compounds **1** (**left**) and **2** (**right**) isolated from *Solidago rugosa* root and leaf, respectively. For fragmentation the collision energy was set to 20 eV.

Isolates **1** and **2** were detectable by HPLC–qTOF-MS in both positive and negative ionization modes as the intense signals of protonated (*m*/*z* 317.2114 and *m*/*z* 303.2325, respectively, [M+H]^+^) and deprotonated (*m*/*z* 315.1965 and *m*/*z* 301.2171, respectively, [M–H]^−^) molecules corresponding to the compounds with the molecular formulae C_20_H_28_O_3_ and C_20_H_30_O_2_, respectively (Figure 3). The presence of the sodium adducts (*m*/*z* 339.1932 and *m*/*z* 325.2139, respectively, [M+Na]^+^) and the sodium adducts of the deprotonated dimer (*m*/*z* 653.3846 and *m*/*z* 625.4284, respectively, [2M–2H+Na]^−^) was also observed. Additionally, the dehydration of protonated molecule of Compound **1** afforded the peak at *m*/*z* 299.2008 [M+H–H_2_O]^+^. In order to verify the assignments, the fragmentation pattern of the protonated molecules was obtained at 20 eV collision energy, resulting in the subsequent losses of H_2_O and HCOOH moieties and the cleavage of the diterpene skeleton. The MS spectrometric data were confirmed by comparing them to the predicted spectrum [31] and to that reported earlier in the literature [32].

The molecular formula of Compound **1** corresponds to seven double bond equivalents. Its ^1^H NMR spectrum in methanol-*d*_4_ indicated the presence of two isolated methyl groups at *δ* 1.28 (s, 3H, H_3_-19) and 0.79 (s, 3H, H_3_-20) ppm, a methyl group adjacent to a methine at *δ* 0.86 (d, *J* = 6.7 Hz, 3H, H_3_-17) ppm, an olefinic proton at *δ* 6.65 (dd, *J* = 4.7, 2.8 Hz, 1H, H-3) ppm as well as three aromatic protons at *δ* 7.38 (t, *J* = 1.7 Hz, 1H, H-15), 7.26 (dt, *J* = 1.7, 0.9 Hz, 1H, H-16), and *δ* 6.29 (dd, *J* = 1.9, 0.9 Hz, 1H, H-14) ppm. The ^13^C NMR spectrum revealed 20 carbon resonances, including two overlapping signals at *δ* 144.0 (C-4, C-15), three methyl carbons at *δ* 21.1 (C-19), 18.8 (C-20) and 16.3 (C-17) ppm, four aromatic carbons at *δ* 144.0 (C-15), 139.7 (C-16), 126.9 (C-13), and 111.9 (C-14) ppm, two olefinic carbons at *δ* 144.0 (C-4) and 138.1 (C-3) ppm as well as one carboxylic carbon at *δ* 171.1 (C-18) ppm. Based on the spectral data along with the 2D homo- and heteronuclear correlations, the structure of Compound **1** was elucidated as hardwickiic acid, a *trans*-clerodane diterpenoid carboxylic acid bearing *β*-substituted furan moiety. (–)-Hardwickiic acid has been isolated from *S. rugosa* by Lu et al. [10,33], which allowed the deduction of its absolute configuration. The levorotatory enantiomer was supported by the observed negative sign of its specific rotation value (${\left[\alpha \right]}_{D}^{25}$ = −104.4 (*c* 0.4775, EtOH)), which is similar to the previously reported data (${\left[\alpha \right]}_{D}^{25}$ = −116.5 (*c* 1.09, EtOH), [34]). Furthermore, due to the lack of the reported NMR spectra recorded in the methanol-*d*_4_ solvent, the published NMR spectroscopic data of its antipode, *ent*-(+)-hardwickiic acid [35], was used for confirmation as enantiomers have identical NMR spectra. The two spectra were in excellent agreement, which corroborated the proposed structure.

The molecular formula of Compound **2** represents six double bond equivalents. The ^1^H NMR spectrum of Compound **2** in methanol-*d*_4_ featured resonances at *δ* 1.24 (s, 3H, H_3_-19) and 0.84 (s, 3H, H_3_-20) ppm, indicating the presence of two isolated methyl groups. Two signals at *δ* 1.02 (d, *J* = 6.9 Hz, 3H, H_3_-16 or H_3_-17) and 1.01 (d, *J* = 6.9 Hz, 3H, H_3_-16 or H_3_-17) ppm were also observed, which can be attributed to two diastereotopic methyl groups adjacent to the same methine. The resonances at *δ* 5.75 (br s, 1H, H-14) and 5.32 (m, 1H, H-7) ppm established the existence of two olefinic protons. The ^13^C NMR spectrum of Compound **2** comprised 20 carbon signals, including four methyl carbons at *δ* 21.8 (C-16 or C-17), 21.3 (C-16 or C-17), 17.5 (C-19), and 14.4 (C-20) ppm, four olefinic carbons at *δ* 145.8 (C-13), 136.8 (C-8), 124.0 (C-14), and 121.5 (C-7) ppm as well as one carboxylic carbon at *δ* 182.5 (C-18) ppm. Based on its 1D and 2D NMR characteristics and the observed negative sign of its specific rotation value (${\left[\alpha \right]}_{D}^{25}$ = −91.4 (*c* 0.1225, EtOH)), the structure of Compound **2** was identified as (–)-abietic acid, a tricyclic abietane diterpenoid carboxylic acid. Comparing the measured spectral data in the methanol-*d*_4_ solvent [36] and the observed specific rotation with the previously published value (${\left[\alpha \right]}_{D}^{22}$ = −101.7 (*c* 0.6, EtOH), [37]), the proposed structure was confirmed. The complete ^1^H and ^13^C NMR resonance assignments of Compounds **1** and **2** are listed in Table 1. The measured 1D and 2D NMR spectra of Compounds **1** and **2** are presented in Figures S1–S30.

(–)-Hardwickiic acid is a common constituent throughout the *Solidago* genus. Apart from *S. rugosa*, it was isolated from *S. arguta* [38], *S. juncea* [39], and *S. serotina* [40]. Moreover, its presence was also described in various families of plants, e.g., *Hardwickia pinnata* [41], *Croton aromaticus* [42], *Grangea maderaspatana* [43], and *Echinodorus grandiflorus* [44]. (–)-Abietic acid is a widespread resin acid in nature initially isolated from rosin and occurring mainly in trees, e.g., in the resin of *Pinus* species (Resina Pini) [45], the leaves of *Pimenta racemosa* var. *grisea* [46], and the cones of *Abies nordmanniana* ssp. *equi-trojani* [47]. However, to the best of our knowledge, we report here for the first time that (–)-abietic acid is also present and abundant in a plant (*S. rugosa*) belonging to the *Asteraceae* family.

The antibacterial experiments revealed that both compounds exhibited similar efficiency against all studied Gram-positive bacterial strains, with approximately 5–20 times higher IC_50_ values (between 1 and 5.1 µg/mL) than that of the positive control gentamicin (Table 2). In the used concentrations, the MBC was reached only in the *B. spizizenii* assay with 10.4 and 5.2 µg/mL for Compounds **1** and **2**, respectively. Weak inhibition was noticed against the Gram-negative *X. arboricola* pv. *pruni*. Both Compounds **1** and **2** exhibited weak activity in the *F. avenaceum* antifungal assay with an IC_50_ value 15 and 33 times higher than that of the reference fungicide benomyl. Compound **1** was also more effective against *B. sorokiniana* with an IC_50_ of 3.8 µg/mL, which means a 20 times stronger antifungal potency when compared to that of benomyl.

(–)-Hardwickiic acid exerted mild cytotoxic activity towards HuCCA-1 (human cholangiocarcinoma cancer), KB (human epidermoid carcinoma of the mouth), HeLa (cervical adenocarcinoma), MDA-MB231 (human breast cells), and T47D (human mammary adenocarcinoma) cell lines with IC_50_ values ranging from 28.0 to 45.0 µg/mL [36]. (–)-Abietic acid possessed moderate cytotoxicity against SK-BR-3 (breast) human cancer cell line with an IC_50_ value of 37.5 µg/mL, although it proved to be inactive against HL60 (leukemia), A549 (lung), and AZ521 (stomach) human cancer lines [48]. (–)-Hardwickiic acid was found antibacterial against several Gram-positive and Gram-negative bacterial strains, including human pathogens (among others, *Enterococcus faecalis*, *Klebsiella aerogenes*, *Pseudomonas aeruginosa*, *Salmonella typhi*, *Shigella flexneri*, *Staphylococcus aureus*, etc.), with IC_50_ values varying from 1.22 to 78.12 µg/mL [49], being comparable or less potent to the Gram-positive bacteria investigated in our study. However, a higher efficiency was observed against Gram-negative bacteria when compared to our findings [49]. The antibacterial activity of (–)-abietic acid was tested on numerous Gram-positive and Gram-negative bacterial strains, including human pathogens (*Acinetobacter baumannii*, *Cutibacterium acnes*, *K. pneumoniae*, *S. aureus*, etc.). In the case of Gram-positive organisms, the antibacterial effects were comparable or weaker than in our experiments [50,51,52,53]. However, it inhibited the growth of Gram-negative bacteria to a greater extent when compared to our results [50,51,52,53]. The antileishmanial effect of (–)-hardwickiic acid against promastigotes of *Leishmania major* and *L. donovani* was evaluated, and the assays provided the IC_50_ values of 62.82 and 31.57 µM, respectively [54]. (–)-Abietic acid displayed a neuroprotective effect against in vitro cerebral ischemia induced by iodoacetic acid treatment using the mouse HT22 hippocampal nerve cell line [55]. The in vitro anti-inflammatory activity of (–)-abietic acid was also demonstrated, as it inhibited the IL-1β-induced production of TNF-α, NO, and PGE2 and suppressed the COX-2 expression in human osteoarthritis chondrocytes [56]. (–)-Abietic acid inhibited the activity of the soybean 5-lipoxygenase enzyme with an IC_50_ value of 29.5 µM, suggesting that it may be used as a therapeutic agent in the treatment of various human diseases, including allergy, asthma, arthritis, and psoriasis [47,57]. The observed antifungal potency of (–)-hardwickiic acid against *F. avenaceum* was similar to its previously reported inhibition against *Candida albicans* and *C. glabrata*, albeit it displayed a stronger activity against *F. avenaceum* than against *C. krusei* [49]. A prominent antifungal inhibitory activity of (–)-abietic acid was reported against *Rhodotorula mucilaginosa* and *Cladosporium cladosporioides* with the MIC_90_ values of 31 and 63 µg/mL, respectively [50], in both cases indicating a stronger effect compared to that against the fungal strains in our work. However, these compounds have not yet been tested against plant pathogens to assess their potential as a basis for a new plant protection agent.

## 3. Materials and Methods

### 3.1. Materials

Glass- and aluminum-backed HPTLC silica gel 60 F_254_ layers were purchased from Merck (Darmstadt, Germany). Isopropyl acetate and gentamicin were from Sigma (Budapest, Hungary). Solvents of analytical grade for HPTLC and flash chromatography were obtained from Molar Chemicals (Halásztelek, Hungary). Vanillin was from Reanal (Budapest, Hungary). 3-(4,5-Dimethylthiazol-2-yl)-2,5-diphenyltetrazolium bromide (MTT) was acquired from Carl Roth (Karlsruhe, Germany), and concentrated sulfuric acid (96%) from Carlo Erba (Milan, Italy). Methanol-*d*_4_ (99.8 atom% D) was purchased from VWR (Budapest, Hungary), and benomyl (Fundazol 50WP) from Chinoin (Budapest, Hungary).

*S. rugosa* Mill. plants, with young shoots, were purchased from Lichtnelke Pflanzenversand (Hamburg, Germany), grown and bred in the greenhouse at the Plant Protection Institute, Centre for Agricultural Research (CAR), Budapest, Hungary. Voucher samples (PPI-MA-Srr-01 and PPI-MA-Srl-01) are available at the Herbarium of Plant Protection Institute, CAR, Budapest, Hungary. The roots and leaves of flowering plants were collected in July 2021. The samples were carefully cleaned with tap water, dried at room temperature for a week, and stored in a cool and dry place until sample preparation.

The Gram-positive *Bacillus subtilis* soil bacterium (strain F1276) was received by József Farkas (Central Food Research Institute, Budapest, Hungary), and the Gram-positive *Bacillus spizizenii* soil bacterium (DSM 618) was from Merck. The Gram-positive bean pathogen *Curtobacterium flaccumfaciens* pv. *flaccumfaciens* (NCAIM B.01609) was purchased from the National Collection of Agricultural and Industrial Microorganisms (NCAIM, Budapest, Hungary). The tomato pathogen *Clavibacter michiganensis* subsp. *michiganensis* strain was isolated from tomato in 1978 (49/1, Sándor Süle, Plant Protection Institute, CAR, Budapest, Hungary). The Gram-negative Hungarian *Xanthomonas arboricola* pv. *pruni* strain was isolated from *Prunus armeniaca* L. cv. Bergecot in 2016 (XapHU1, I. Schwarczinger, Plant Protection Institute, CAR, Budapest, Hungary) [58]. *Fusarium avenaceum* strain IMI 319947 was from CABI-IMI Culture Collection, Egham, UK, and *Bipolaris sorokiniana* (Sacc.) Shoemaker H-299 (NCBI GenBank accession No. MH697869) was collected from barley in 2008 in Hungary.

### 3.2. Extraction and Isolation

Powdered (Sencor SCG 2050RD, Říčany, Czech Republic) samples of *S. rugosa* were macerated in ethanol (150 mg/mL) for 24 h, and the filtered crude extracts were analyzed by HPTLC. For isolation, the dried and ground roots (18.3 g) and leaves (10.2 g) were separately extracted with 3 × 300 mL ethanol by maceration for 24 h. Following filtration (Whatman No. 2 filter paper, Sigma), the extracts were combined, and dried by a rotary evaporator (Büchi Rotavapor R-134). The dry residue of roots (173 mg) and leaves (266 mg) was re-suspended in 3 mL of CHCl_3_ using ultrasonication. The whole extracts were subjected to flash chromatography (CombiFlash NextGen 300, Teledyne Isco, Lincoln, NE, USA) on a silica gel column (RediSep Rf Gold, 20–40 μm, 12 g) using a gradient system of *n*-hexane and ethyl acetate (0–30% in 10 min; 20 mL/min) that provided root (R1–R16) and leaf (L1–L20) fractions. The bioactive fractions R10–R13 (52.7 mg dry weight, Rt = 7.5–8.3 min), as well as L11–L13 (37.4 mg dry weight, Rt = 7.8–8.4 min) with similar compositions, were combined, dried, dissolved in 3 mL chloroform, and further fractionated on a C_18_ column (RediSep Rf Gold, 20–40 μm, 30 g) using a gradient system of water with 0.1% formic acid and methanol (0–14 min: 50–100%, 14–20 min: 100%; 20 mL/min). The main compounds of roots (at Rt = 15.2–16.1 min, fractions 26–27 of 36) and leaves (at Rt = 16.8–17.7 min, fractions 29–30 of 33) were further purified by normal phase flash chromatography (RediSep Rf Gold, 20–40 μm, 12 g; *n*-hexane–acetone 0–100% in 17 min; 20 mL/min) to obtain Compounds **1** (25.5 mg) and **2** (9.4 mg), respectively. Fractions were monitored after each step with HPTLC hyphenations (see below).

### 3.3. High-Performance Thin-Layer Chromatography Hyphenations

Crude extracts (150 mg plant material/mL, 2 µL), flash fractions (3 mg dry residue/mL, 5–10 µL), and isolated compounds in ethanol (2 mg/mL, 0.2–0.5 µL) were applied onto the HPTLC layer by the Automatic TLC Sampler 3 (ATS3, CAMAG, Muttenz, Switzerland), or a 10 µL microsyringe (Hamilton, Bonaduz, Switzerland), as 5 mm bands with 8–10 mm distance between the bands, and 8 mm distance from the lower edge. HPTLC separation was performed with *n*-hexane–isopropyl acetate 4:1 *v*/*v* in a Twin Trough Chamber (20 cm × 10 cm, CAMAG) up to 70 mm from the lower edge of the layer. The segments of the dried chromatograms were documented by a digital camera (Cybershot DSC-HX60, Sony, Neu-Isenburg, Germany) under a UV lamp (CAMAG) at 254 nm or visible light after derivatization with vanillin–sulfuric acid reagent (400 mg vanillin, 100 mL ethanol, and 2 mL concentrated sulfuric acid; heating at 110 °C for 5 min), or a bioassay.

The preparation of the *B. subtilis* cell suspension and the performance of the direct bioautographic assay were described previously [59]. The test bacterium was grown in lysogeny broth (LB: 10 g/L tryptone (Reanal), 5 g/L yeast extract (Scharlau, Barcelona, Spain), and 10 g/L sodium chloride (Reanal)) at 37 °C on an orbital shaker (120 rpm) to reach late exponential phase (OD_600_ = 1.2). The steps of the direct bioautography were: 1. immersion of the developed and dried HPTLC plates into the bacterial cell suspension; 2. incubation of the bioautogram for 2 h, at 37 °C and 100% humidity; 3. dipping of the bioautogram into a vital dye solution (MTT, 1 mg/mL in water); 4. further incubation for 30 min; 5. documentation of the bioautogram under visible light (bright zones against a purple background indicate the antibacterial compounds).

### 3.4. HPLC–ESI-qTOFMS

HPLC-MS analyses were performed by an Agilent 1200 Series HPLC system coupled to an Agilent 6520A qTOF-MS equipped with an electrospray ionization (ESI) probe (Agilent Technologies, Santa Clara, CA, USA). For the separation, a Zorbax Eclipse XDB-C18 Solvent Saver Plus reversed-phase column (75 × 3.0 mm i.d.; 3.5 µm; Agilent Technologies, Santa Clara, CA, USA) was employed. The mobile phase consisted of water with 0.1% formic acid and 5% acetonitrile (A) and acetonitrile with 0.1% formic acid and 5% water (B). The elution was carried out at 25 °C with a flow rate of 0.5 mL/min using the following linear gradient: 0–100% B (0–10 min), 100% B (10–12 min), and 0% B (12–15 min). The injection volume was 1 µL. Analytes were detected using drying gas (nitrogen) at 350 °C and 12 L/min and nebulizer gas at 40 psi. For collision-induced dissociation (CID), the parameters were as follows: collision gas: high-purity nitrogen; collision energy: 10–40 eV; fragmentor voltage: 110 V; capillary voltage: 3500 V. MS and MS/MS spectra were acquired in the *m*/*z* range of 25–700 and 45–600, respectively. Reference masses of *m*/*z* 121.050873 and 922.009798 for positive and *m*/*z* 112.985587 and 1033.988109 for negative ionization were applied in the internal calibration. The collected data were evaluated with MassHunter Qualitative Analysis 10.0 software (Agilent Technologies, Santa Clara, CA, USA).

### 3.5. NMR Spectroscopy

The NMR samples were prepared by dissolving the isolated Compounds **1** and **2** in 0.6 mL of methanol-*d*_4_ and were transferred to a standard 5 mm NMR tube for analyses. NMR spectra were recorded on a Varian DDR 600 (^1^H: 599.9 MHz, ^13^C: 150.9 MHz; 14.1 T) spectrometer equipped with a dual 5 mm inverse-detection pulsed-field gradient (IDPFG) probehead at 298 K. VnmrJ 3.2C software was utilized for instrument operation and instrument control as well as data acquisition and data processing. All applied pulse sequences were part of the Chempack 5.1 standard pulse program library of the spectrometer. ^1^H and ^13^C chemical shifts (*δ*) are given on the *δ*-scale, reported in ppm, and referenced to the applied NMR solvent (CHD_2_OD residual peak at *δ*(^1^H) 3.31 ppm and CD_3_OD at *δ*(^13^C) 49.0 ppm), whereas spin–spin coupling constants (*J*) are provided in Hz. The complete resonance assignments were established from comprehensive one-(^1^H and ^13^C) and two-dimensional homonuclear (^1^H–^1^H COSY, ^1^H–^1^H TOCSY, and ^1^H–^1^H NOESY) and heteronuclear (^1^H–^13^C edHSQC (^1^*J*_C–H_ = 140 Hz) and ^1^H–^13^C HMBC (^*n*^*J*_C–H_ = 8 Hz), both of them gradient-enhanced with adiabatic pulses) NMR experiments. To achieve the appropriate resolution in the 2D heteronuclear measurements for Compound **2**, band-selective HSQC (bsHSQC) and HMBC (bsHMBC) spectra were also collected.

### 3.6. Determination of Optical Rotations

Optical rotations of the isolated compounds were measured at 25 °C with a Perkin Elmer 341 LC polarimeter (Waltham, MA, USA) in ethanol (**1**—0.4775 g/100 mL; and **2**—0.1225 g/100 mL) at 589.3 nm (D-line of sodium) with an optical path length of 100 mm and an integration time of 2 s.

### 3.7. Microdilution Assays

*B. spizizenii* and *X. arboricola* pv. *pruni* were grown by shaking at 120 rpm in LB at 37 °C and 28 °C, respectively. *C. flaccumfaciens* pv. *flaccumfaciens* and *C. michiganensis* ssp. *michiganensis* were grown in Nutrient Broth (16 g/L Nutrient Broth (Biolab, Budapest, Hungary)) by shaking at 120 rpm at 28 °C. Liquid cultures of both fungi, *B. sorokiniana* and *F. avenaceum*, were shaken in LB at 120 rpm, 21 °C, in the dark, for three days. Mycelia were collected, washed in fresh LB, then fragmented using a homogenizer (FastPrep^®^-24 Classic, MP Biomedicals, Beograd, Serbia) by putting the mycelia in 1 mL of the same medium into 2 mL Eppendorf tubes containing 7 × 2 mm glass beads. The homogenizer’s acceleration was 4.5 m/s, and the duration 20 s.

We determined the MBC, MIC, and IC_50_ of the isolated Compounds **1** (10 mg/mL in ethanol) and **2** (5 mg/mL in ethanol) using 96-well microplates. Gentamicin (0.1 mg/mL in ethanol) or benomyl (25 mg/mL in ethanol) served as the positive and ethanol as the negative control. A two-fold dilution series starting from 5 µL of each isolate was prepared in ethanol in the wells. When the ethanol evaporated under a sterile laminar airflow, 150 µL of a bacterial suspension (10^5^ CFU/mL in LB) or 120 µL of a mycelium suspension (OD_600_ = 0.1 in LB) was added to each well. Starting values of the absorbance at 600 nm were measured by a microplate spectrophotometer (Labsystems Multiscan MS 4.0, Thermo Fisher Scientific, Budapest, Hungary). The bacterial cultures were shaken at 900 rpm and an appropriate temperature with a microplate shaker (Grant PHMP) for 24 h, while fungal cultures were kept at 21 °C for 72 h. Then, the OD_600_ values were again observed. The experiment was repeated twice with three parallels, and the results were averaged. The MBC was determined by plotting 5 µL of each well onto appropriate agar layers, and after incubation, by checking the presence or absence of bacterial colonies.

## 4. Conclusions

Two antimicrobial diterpenes were detected and isolated from the roots and leaves of *S. rugosa* utilizing a bioassay-guided process. Both compounds were present in both tissues, but Compound **1** was dominant in the roots, and Compound **2** in the leaves. These compounds, identified as (–)-hardwickiic acid (**1**) and (–)-abietic acid (**2**), exhibited notable antimicrobial activity against Gram-positive bacterial and fungal strains, including plant pathogens. The results imply that suitable structural modification and formulation could makethese compounds promising agrochemical agents.

## Figures and Tables

**Table 1:** ^1^H and ^13^C NMR (CD_3_OD, 600/151 MHz) resonance assignments of (–)-hardwickiic acid (**1**) and (–)-abietic acid (**2**).

	(–)-Hardwickiic acid (**1**) 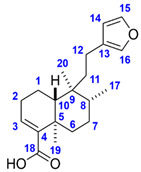		(–)-Abietic acid (**2**) 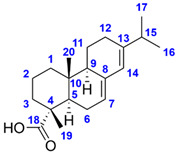	
**#**	**^1^H *δ* (ppm)**	**^13^C *δ* (ppm)**	**^1^H *δ* (ppm)**	**^13^C *δ* (ppm)**
**1a**	1.53 (m, 1H)	18.6	1.15 (m, 1H)	39.7
**1b**	1.72 (m, 1H)		1.90 (ov., 1H)	
**2a**	2.18 (m, 1H)	28.1	1.57 (m, 2H)	19.2
**2b**	2.30 (m, 1H)			
**3a**	6.65 (dd, *J* = 4.7, 2.8 Hz, 1H)	138.1	1.63 (m, 1H)	38.6
**3b**			1.79 (ov., 1H)	
**4**	–	144.0	–	47.4
**5**	–	38.7	2.06 (ov., 1H)	46.5
**6a**	2.39 (dt, *J* = 13.1, 3.4 Hz, 1H)	37.2	1.81 (ov., 1H)	26.7
**6b**	1.15 (td, *J* = 13.1, 3.9 Hz, 1H)		2.06 (ov., 1H)	
**7a**	1.52 (m, 1H)	28.5	5.32 (m, 1H)	121.5
**7b**	1.42 (m, 1H)			
**8**	1.62 (m, 1H)	37.6	–	136.8
**9**	–	40.0	1.89 (ov., 1H)	52.6
**10**	1.41 (m, 1H)	48.1	–	35.6
**11a**	1.56 (m, 1H)	40.2	1.18 (ov., 1H)	23.7
**11b**	1.69 (m, 1H)		1.82 (ov., 1H)	
**12a**	2.22 (m, 1H)	19.1	2.07 (m, 2H)	28.3
**12b**	2.33 (dd, *J* = 13.7, 4.8 Hz, 1H)			
**13**	–	126.9	–	145.8
**14**	6.29 (dd, *J* = 1.9, 0.9 Hz, 1H)	111.9	5.75 (br s, 1H)	124.0
**15**	7.38 (t, *J* = 1.7 Hz, 1H)	144.0	2.22 (sept, *J* = 6.9 Hz, 1H)	36.2
**16**	7.26 (dt, *J* = 1.7, 0.9 Hz, 1H)	139.7	1.02 (d, *J* = 6.9 Hz, 3H) *	21.3 *
**17**	0.86 (d, *J* = 6.7 Hz, 3H)	16.3	1.01 (d, *J* = 6.9 Hz, 3H) *	21.8 *
**18**	–	171.1	–	182.5
**19**	1.28 (s, 3H)	21.1	1.24 (s, 3H)	17.5
**20**	0.79 (s, 3H)	18.8	0.84 (s, 3H)	14.4

* interchangeable resonances (diastereotopic methyl groups); ov.: overlapping peaks.

**Table 2:** The half maximal inhibitory concentration (IC_50_), minimal inhibitory concentration (MIC), and minimal bactericidal concentration (MBC) values of isolates and two positive controls in µg/mL against four bacterial and two fungal strains as compared to gentamicin and benomyl.

	(–)-Hardwickiic Acid (Compound 1)			(–)-Abietic Acid (Compound 2)			Gentamicin			Benomyl	
Strain	IC_50_	MIC	MBC	IC_50_	MIC	MBC	IC_50_	MIC	MBC	IC_50_	MIC
1	1.0 ± 0.1	10.4	10.4	3.6 ± 0.1	5.2	5.2	0.16 ± 0.01	3.33	3.33		
2	5.1 ± 0.2	33.3	>333	2.3 ± 0.1	8.3	>333	0.37 ± 0.03	1.7	1.7		
3	2.0 ± 0.1	2.6	>333	2.0 ± 0.1	2.6	>333	0.33 ± 0.01	0.83	1.67		
4	201.2 ± 2.1	>333		166.6 ± 6.8	>333		2.12 ± 0.02	3.3	3.3		
5	73.5 ± 5.0	>417		165.5 ± 13.0	>417					5.1 ± 2.9	520.8
6	3.8 ± 0.2	>417		120.6 ± 8.1	>417					80.4 ± 5.2	>1041.6

Strains: (1) *Bacillus spizizenii* (Gram +), (2) *Clavibacter michiganensis* subsp. *michiganensis* (Gram +), (3) *Curtobacterium flaccumfaciens* pv. *flaccumfaciens* (Gram +), (4) *Xanthomonas arboricola* pv. *pruni* (Gram –), (5) *Fusarium avenaceum*, (6) *Bipolaris sorokiniana*.

## Data Availability

Data are available upon reasonable request.

## Supplementary Materials

The following supporting information can be downloaded at: https://www.mdpi.com/article/10.3390/molecules28093790/s1, Figures S1–S14: NMR spectra of (–)-hardwickiic acid (Compound **1**); Figures S15–S30: NMR spectra of (–)-abietic acid (Compound **2**).

## Sample Availability

Samples of the Compounds **1** and **2** are available from the authors.
