# Supplementary material for: Antimicrobial Diterpenes from Rough Goldenrod (Solidago rugosa Mill.)
Source: Molecules. 2023 Apr 28;28(9):3790. doi: 10.3390/molecules28093790 (PMC10180332; doi:10.3390/molecules28093790)
Supplement: Supplementary file 1 [file molecules-28-03790-s001.zip › molecules-2347020-supplementary.pdf]

# Supplementary Material

## Antimicrobial Diterpenes from Rough Goldenrod (*Solidago rugosa* Mill.)

Márton Baglyas[1,2], Péter G. Ott[1], Ildikó Schwarczinger[1], Judit Kolozsváriné Nagy[1], András Darcsi[3], József Bakonyi[1], Ágnes M. Móricz[1,*]

[1]Plant Protection Institute, Centre for Agricultural Research, ELKH, Herman O. Str. 15, 1022 Budapest, Hungary

[2]Doctoral School of Pharmaceutical Sciences, Semmelweis University, Hőgyes E. Str. 7–9, 1092 Budapest, Hungary

[3]Pharmaceutical Chemistry and Technology Department, National Institute of Pharmacy and Nutrition, Szabolcs Str. 33, 1135 Budapest, Hungary

* Correspondence: moricz.agnes@atk.hu

# Table of contents


| No. | Legend | Page |
|---|---|---|
| **Figure S1.** | $^{1}$H NMR spectrum of (–)-hardwickiic acid (**1**) ($CD_3OD$, 600 MHz). | S-4 |
| **Figure S2.** | $^{1}$H NMR spectrum of (–)-hardwickiic acid (**1**) ($CD_3OD$, 600 MHz, aliphatic region). | S-5 |
| **Figure S3.** | $^{13}$C NMR spectrum of (–)-hardwickiic acid (**1**) ($CD_3OD$, 151 MHz). | S-6 |
| **Figure S4.** | $^{13}$C NMR spectrum of (–)-hardwickiic acid (**1**) ($CD_3OD$, 151 MHz, aliphatic region). | S-7 |
| **Figure S5.** | $^{1}$H–$^{1}$H COSY NMR spectrum of (–)-hardwickiic acid (**1**) ($CD_3OD$, 600 MHz). | S-8 |
| **Figure S6.** | $^{1}$H–$^{1}$H COSY NMR spectrum of (–)-hardwickiic acid (**1**) ($CD_3OD$, 600 MHz, aliphatic region). | S-9 |
| **Figure S7.** | $^{1}$H–$^{13}$C edHSQC NMR spectrum of (–)-hardwickiic acid (**1**) ($CD_3OD$, 600 and 151 MHz). | S-10 |
| **Figure S8.** | $^{1}$H–$^{13}$C edHSQC NMR spectrum of (–)-hardwickiic acid (**1**) ($CD_3OD$, 600 and 151 MHz, aliphatic region). | S-11 |
| **Figure S9.** | $^{1}$H–$^{13}$C HMBC NMR spectrum of (–)-hardwickiic acid (**1**) ($CD_3OD$, 600 and 151 MHz). | S-12 |
| **Figure S10.** | $^{1}$H–$^{13}$C HMBC NMR spectrum of (–)-hardwickiic acid (**1**) ($CD_3OD$, 600 and 151 MHz, aliphatic region). | S-13 |
| **Figure S11.** | $^{1}$H–$^{1}$H TOCSY NMR spectrum of (–)-hardwickiic acid (**1**) ($CD_3OD$, 600 MHz). | S-14 |
| **Figure S12.** | $^{1}$H–$^{1}$H TOCSY NMR spectrum of (–)-hardwickiic acid (**1**) ($CD_3OD$, 600 MHz, aliphatic region). | S-15 |
| **Figure S13.** | $^{1}$H–$^{1}$H NOESY NMR spectrum of (–)-hardwickiic acid (**1**) ($CD_3OD$, 600 MHz). | S-16 |
| **Figure S14.** | $^{1}$H–$^{1}$H NOESY NMR spectrum of (–)-hardwickiic acid (**1**) ($CD_3OD$, 600 MHz, aliphatic region). | S-17 |
| **Figure S15.** | $^{1}$H NMR spectrum of (–)-abietic acid (**2**) ($CD_3OD$, 600 MHz). | S-18 |
| **Figure S16.** | $^{1}$H NMR spectrum of (–)-abietic acid (**2**) ($CD_3OD$, 600 MHz, aliphatic region). | S-19 |
| **Figure S17.** | $^{13}$C NMR spectrum of (–)-abietic acid (**2**) ($CD_3OD$, 151 MHz). | S-20 |
| **Figure S18.** | $^{13}$C NMR spectrum of (–)-abietic acid (**2**) ($CD_3OD$, 151 MHz, aliphatic region). | S-21 |
| **Figure S19.** | $^{1}$H–$^{1}$H COSY NMR spectrum of (–)-abietic acid (**2**) ($CD_3OD$, 600 MHz). | S-22 |
| **Figure S20.** | $^{1}$H–$^{1}$H COSY NMR spectrum of (–)-abietic acid (**2**) ($CD_3OD$, 600 MHz, aliphatic region). | S-23 |
| **Figure S21.** | $^{1}$H–$^{13}$C edHSQC NMR spectrum of (–)-abietic acid (**2**) ($CD_3OD$, 600 and 151 MHz). | S-24 |
| **Figure S22.** | $^{1}$H–$^{13}$C edHSQC NMR spectrum of (–)-abietic acid (**2**) ($CD_3OD$, 600 and 151 MHz, aliphatic region). | S-25 |
| **Figure S23.** | $^{1}$H–$^{13}$C bsHSQC NMR spectrum of (–)-abietic acid (**2**) ($CD_3OD$, 600 and 151 MHz). | S-26 |
| **Figure S24.** | $^{1}$H–$^{13}$C bsHSQC NMR spectrum of (–)-abietic acid (**2**) ($CD_3OD$, 600 and 151 MHz, aliphatic region). | S-27 |
| **Figure S25.** | $^{1}$H–$^{13}$C HMBC NMR spectrum of (–)-abietic acid (**2**) ($CD_3OD$, 600 and 151 MHz). | S-28 |

| | | |
|---|---|---|
| **Figure S26.** | $^{1}H$–$^{13}C$ HMBC NMR spectrum of (–)-abietic acid (**2**) ($CD_3OD$, 600 and 151 MHz, aliphatic region). | S-29 |
| **Figure S27.** | $^{1}H$–$^{13}C$ bsHMBC NMR spectrum of (–)-abietic acid (**2**) ($CD_3OD$, 600 and 151 MHz). | S-30 |
| **Figure S28.** | $^{1}H$–$^{13}C$ bsHMBC NMR spectrum of (–)-abietic acid (**2**) ($CD_3OD$, 600 and 151 MHz, aliphatic region). | S-31 |
| **Figure S29.** | $^{1}H$–$^{1}H$ TOCSY NMR spectrum of (–)-abietic acid (**2**) ($CD_3OD$, 600 MHz). | S-32 |
| **Figure S30.** | $^{1}H$–$^{1}H$ TOCSY NMR spectrum of (–)-abietic acid (**2**) ($CD_3OD$, 600 MHz, aliphatic region). | S-33 |

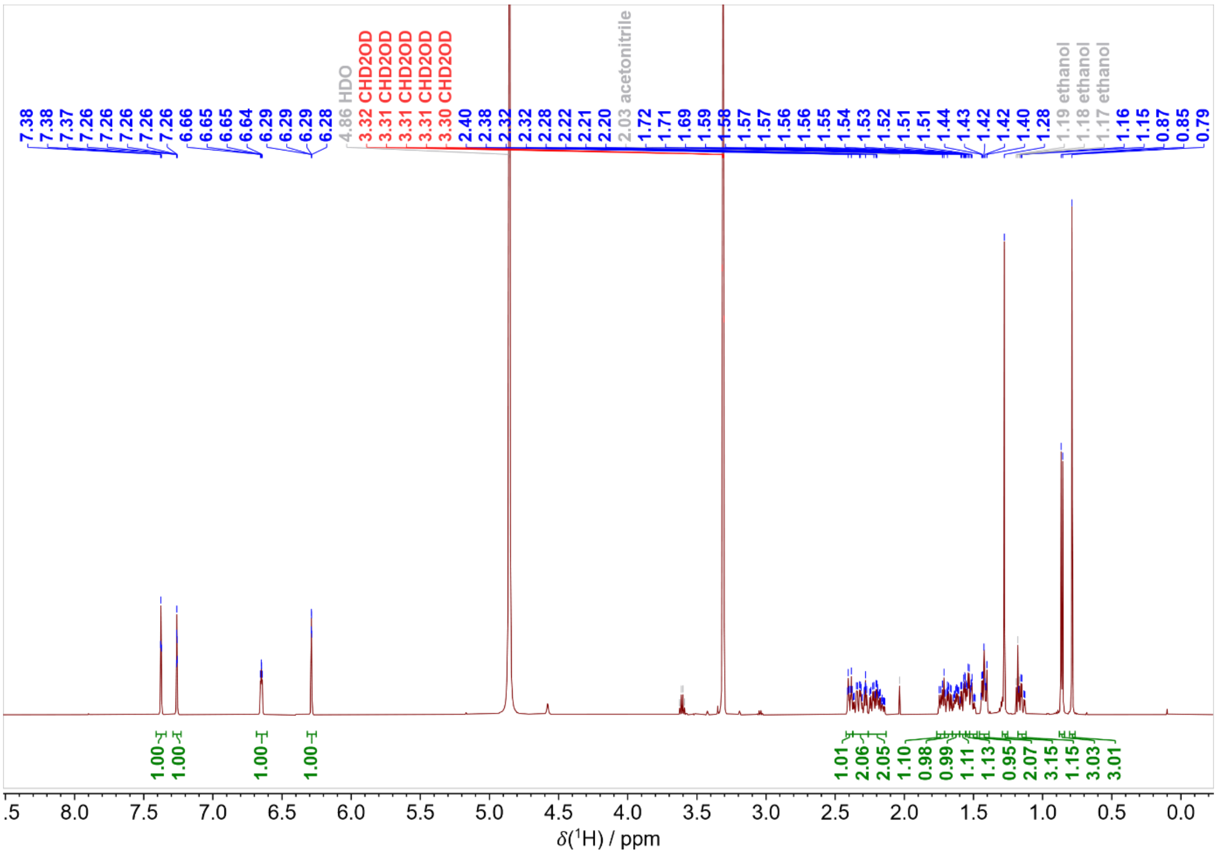

**Figure S1.** $^{1}$H NMR spectrum of (–)-hardwickiic acid (**1**) ($CD_3OD$, 600 MHz).

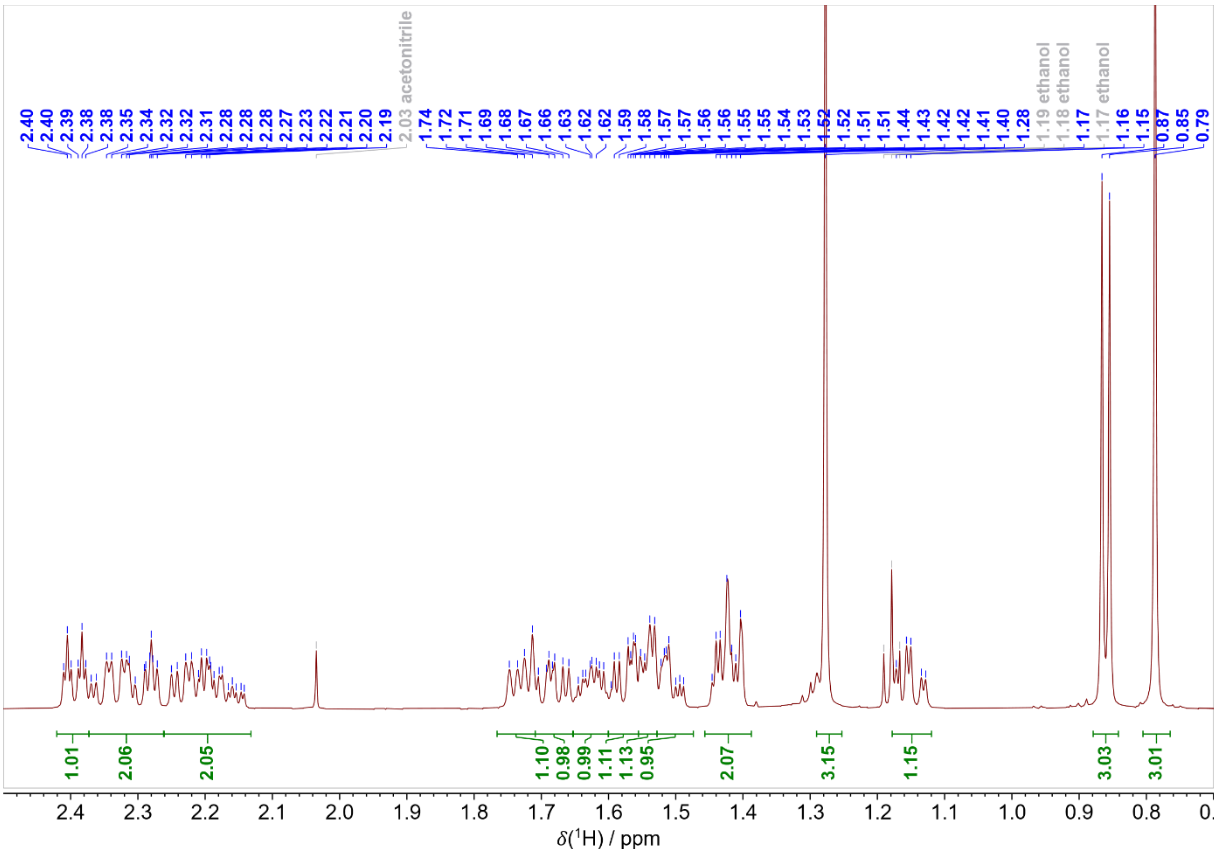

**Figure S2.** $^{1}$H NMR spectrum of (–)-hardwickiic acid (**1**) ($CD_3OD$, 600 MHz, aliphatic region).

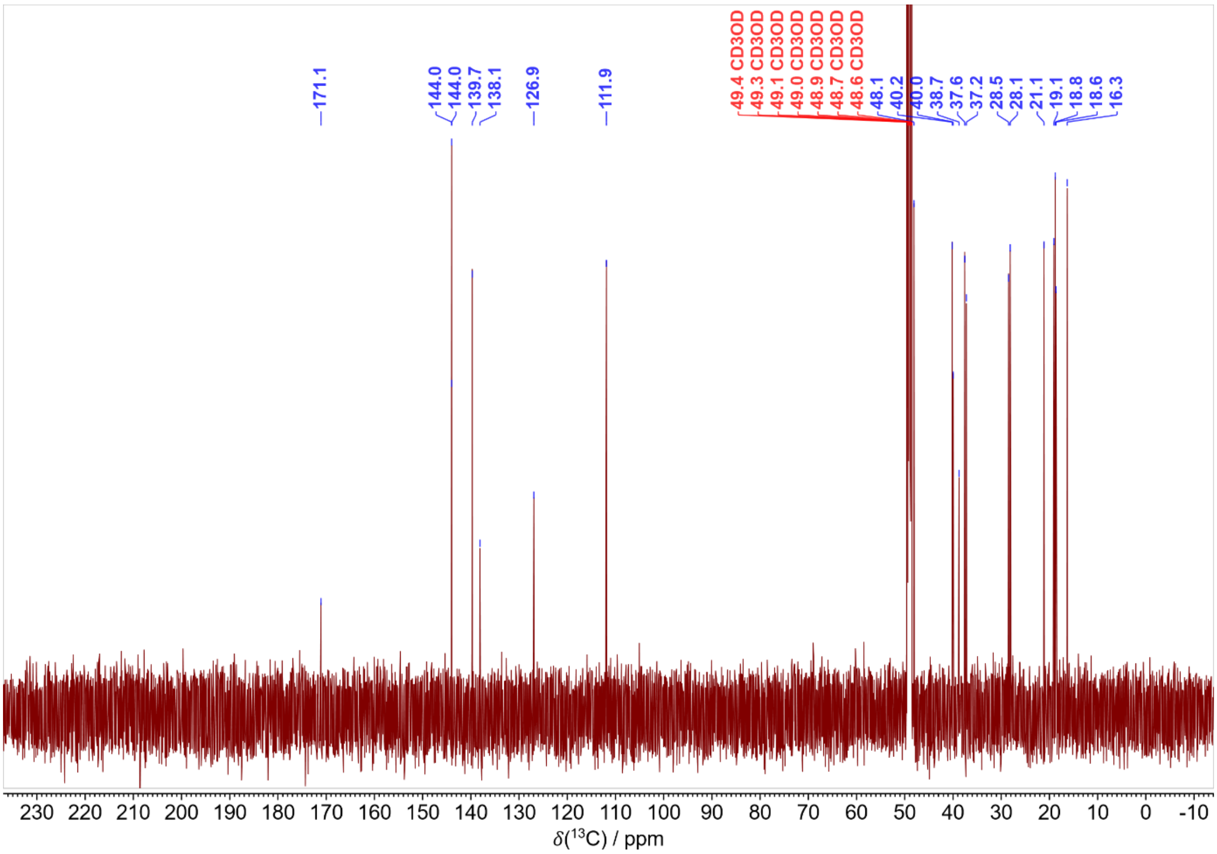


**Figure S3.** $^{13}C$ NMR spectrum of (–)-hardwickiic acid (**1**) ($CD_3OD$, 151 MHz).

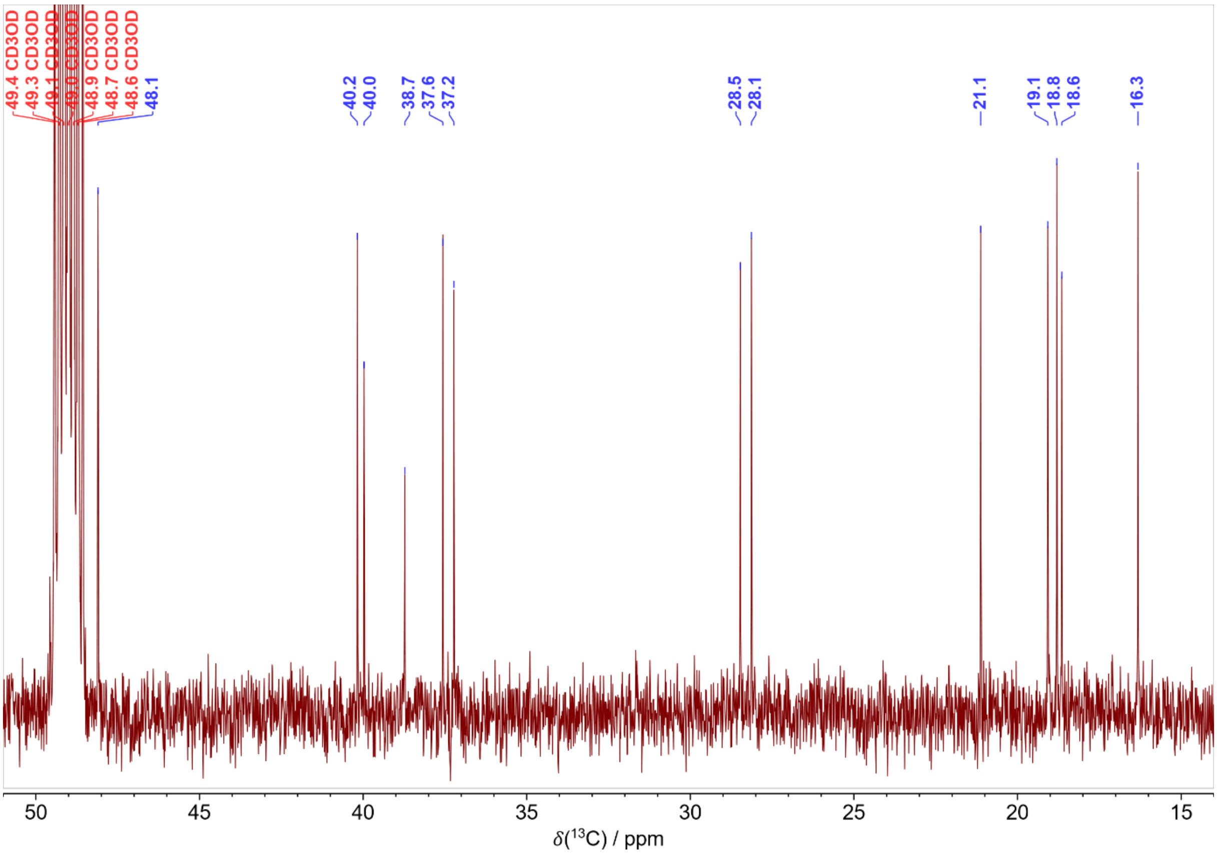


**Figure S4.** $^{13}C$ NMR spectrum of (–)-hardwickiic acid (**1**) ($CD_3OD$, 151 MHz, aliphatic region).

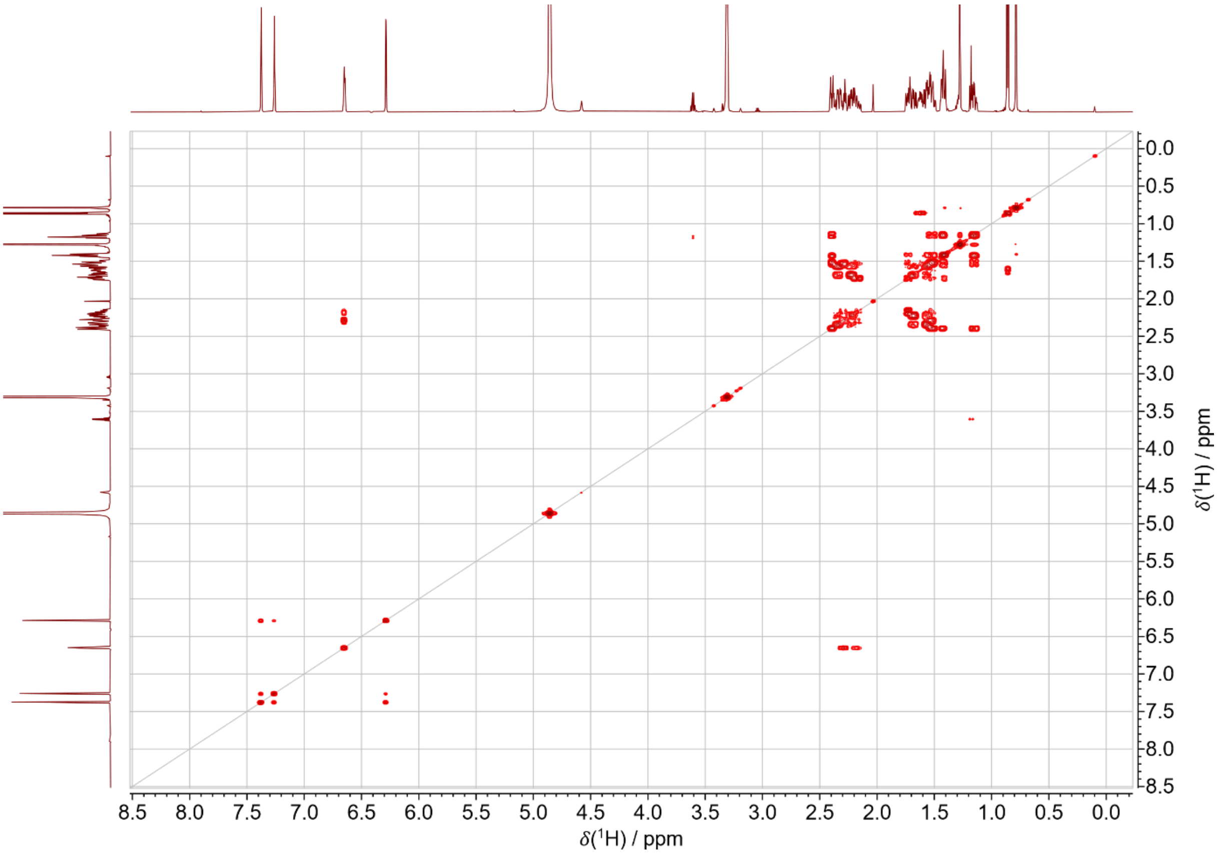


**Figure S5.** $^{1}H$–$^{1}H$ COSY NMR spectrum of (–)-hardwickiic acid (**1**) ($CD_3OD$, 600 MHz).

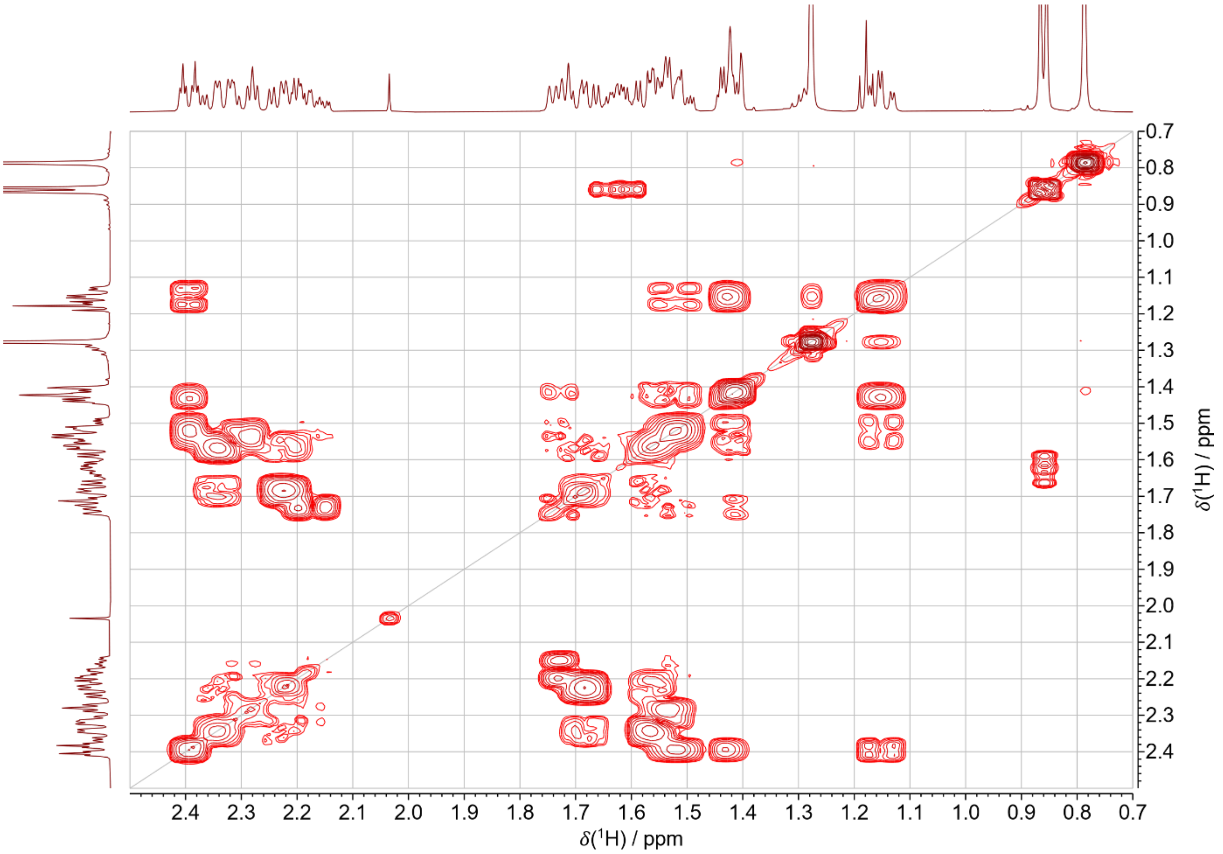


**Figure S6.** $^{1}H$–$^{1}H$ COSY NMR spectrum of (–)-hardwickiic acid (**1**) ($CD_3OD$, 600 MHz, aliphatic region).

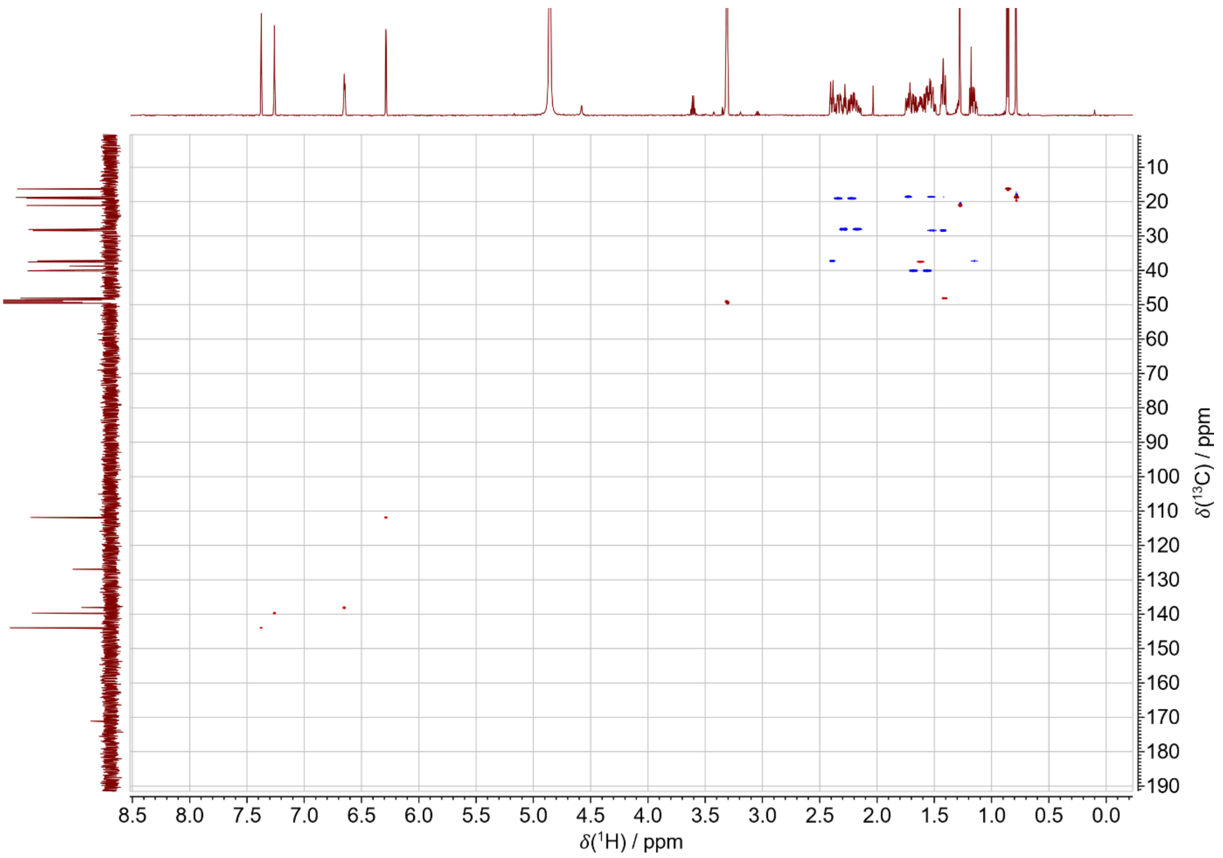


**Figure S7.** $^{1}$H–$^{13}$C edHSQC NMR spectrum of (–)-hardwickiic acid (**1**) ($CD_3OD$, 600 and 151 MHz).

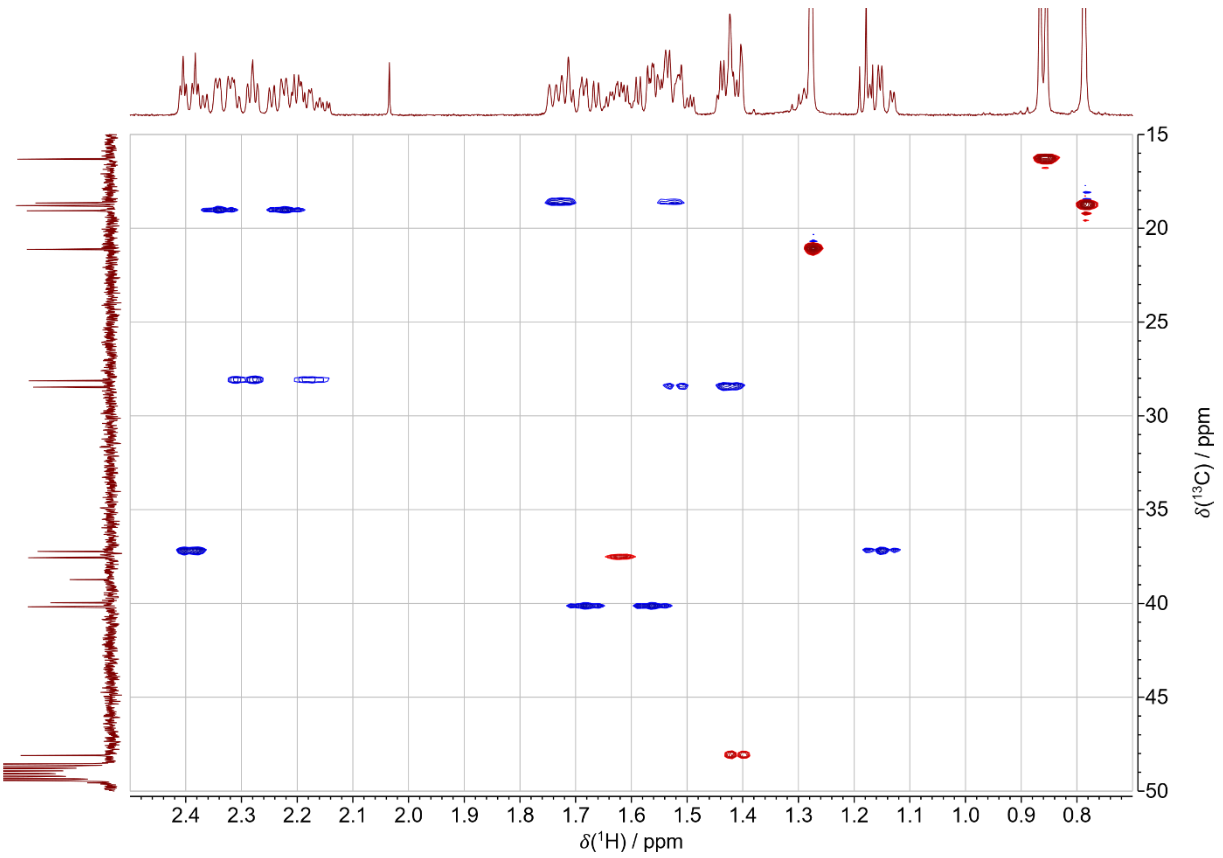


**Figure S8.** ¹H–¹³C edHSQC NMR spectrum of (–)-hardwickiic acid (**1**) ($CD_3OD$, 600 and 151 MHz, aliphatic region).

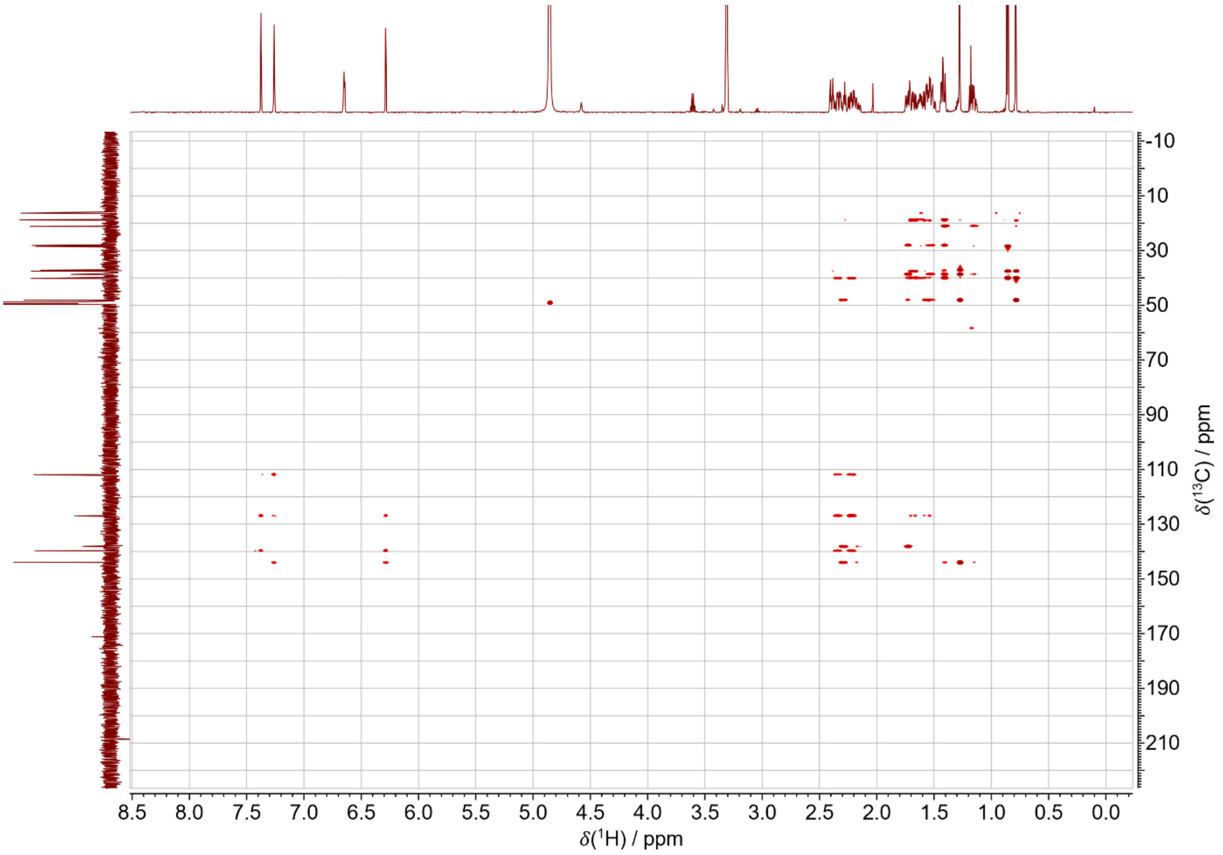


**Figure S9.** $^{1}H$–$^{13}C$ HMBC NMR spectrum of (–)-hardwickiic acid (**1**) ($CD_3OD$, 600 and 151 MHz).

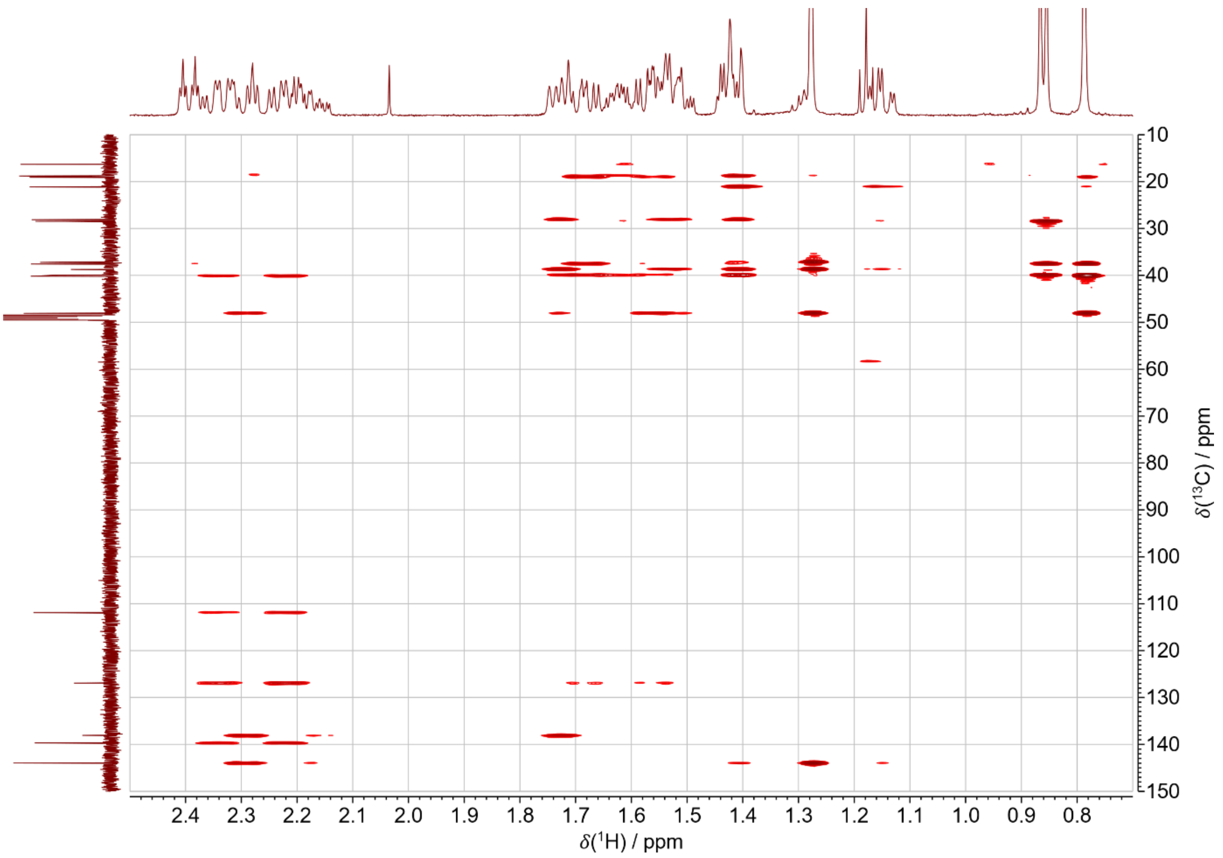


**Figure S10.** $^{1}H$–$^{13}C$ HMBC NMR spectrum of (–)-hardwickiic acid (**1**) ($CD_3OD$, 600 and 151 MHz, aliphatic region).

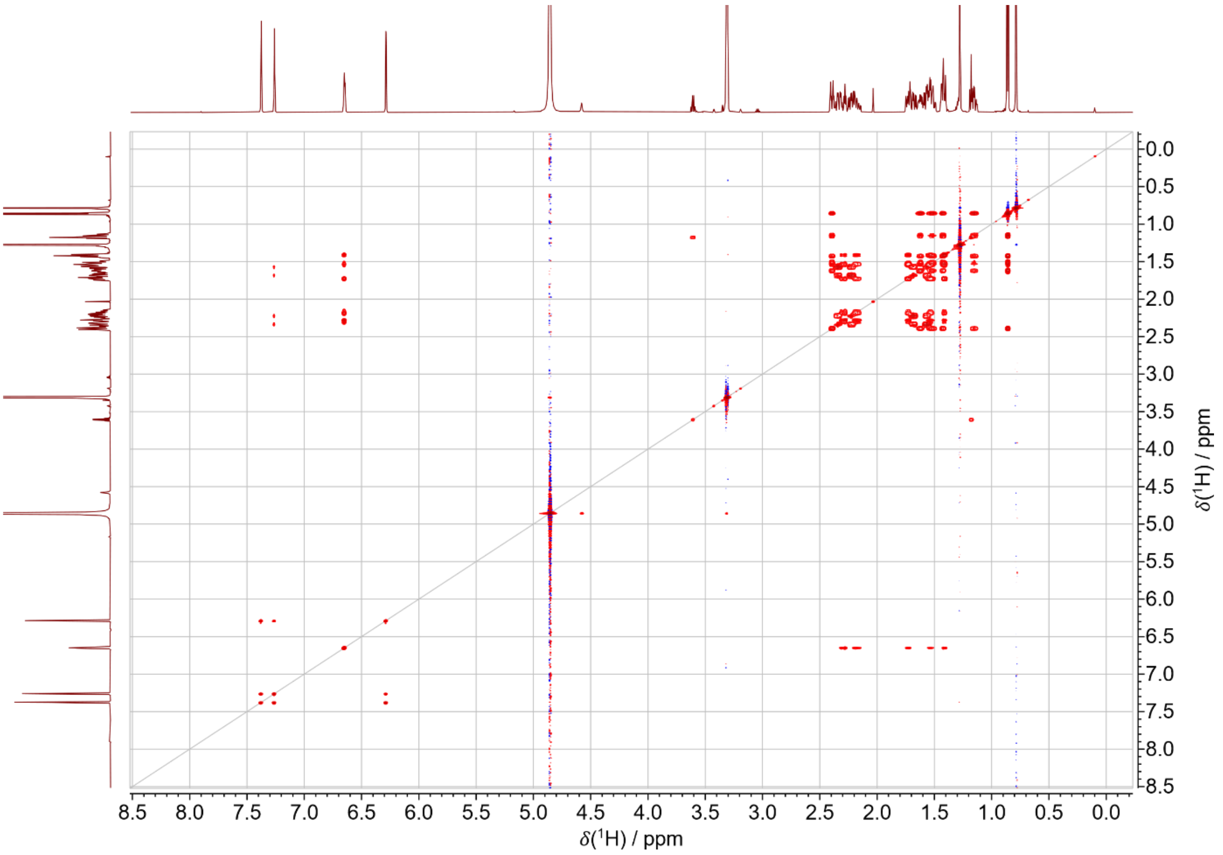


**Figure S11.** $^{1}H$–$^{1}H$ TOCSY NMR spectrum of (–)-hardwickiic acid (**1**) ($CD_3OD$, 600 MHz).

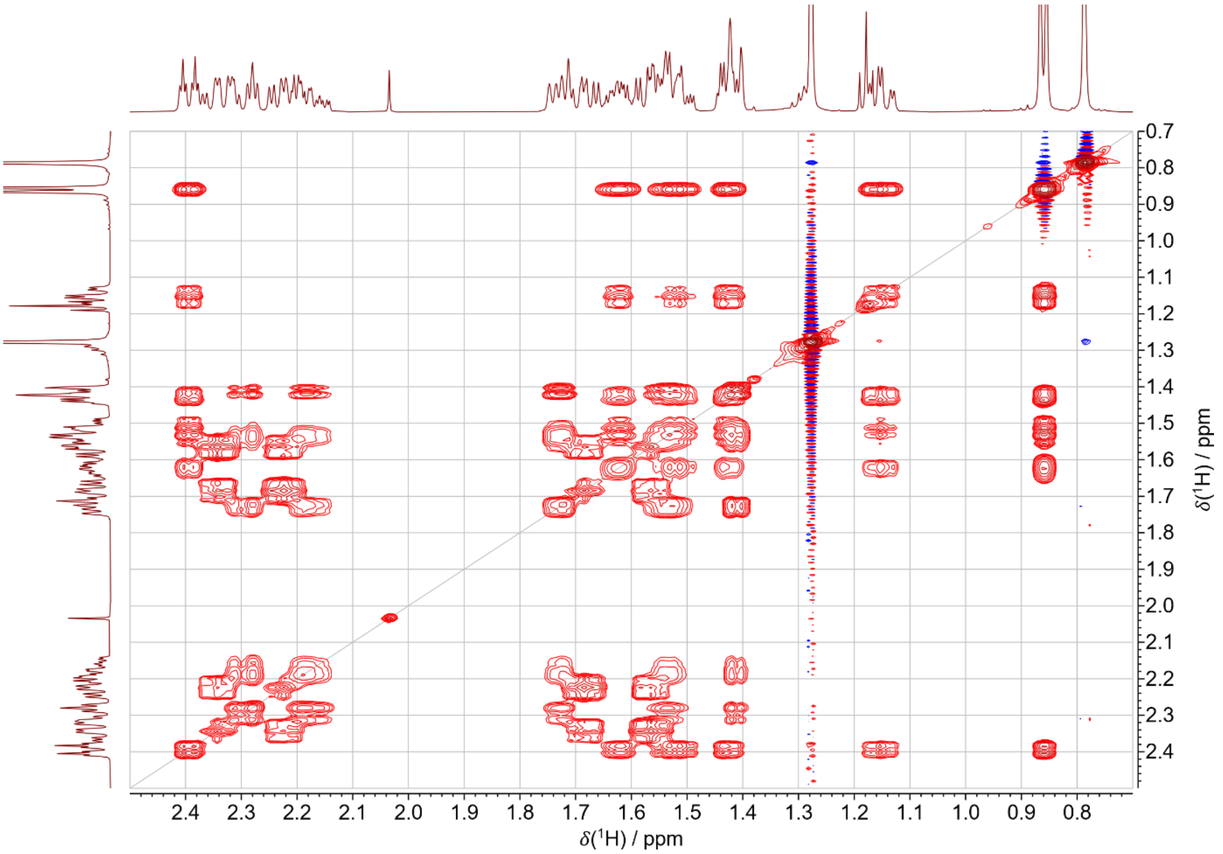


**Figure S12.** $^1H$–$^1H$ TOCSY NMR spectrum of (–)-hardwickiic acid (**1**) ($CD_3OD$, 600 MHz, aliphatic region).

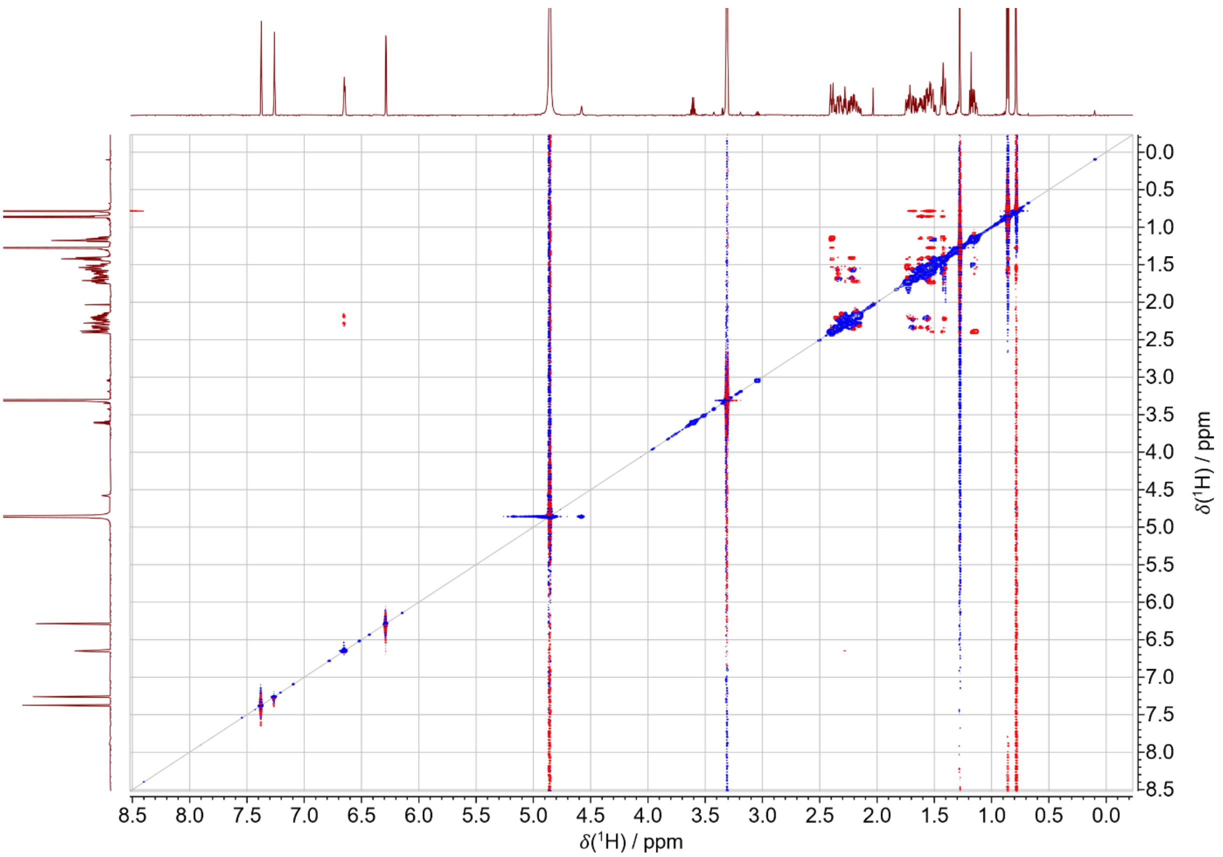


**Figure S13.** $^{1}H$–$^{1}H$ NOESY NMR spectrum of (–)-hardwickiic acid (**1**) ($CD_3OD$, 600 MHz).

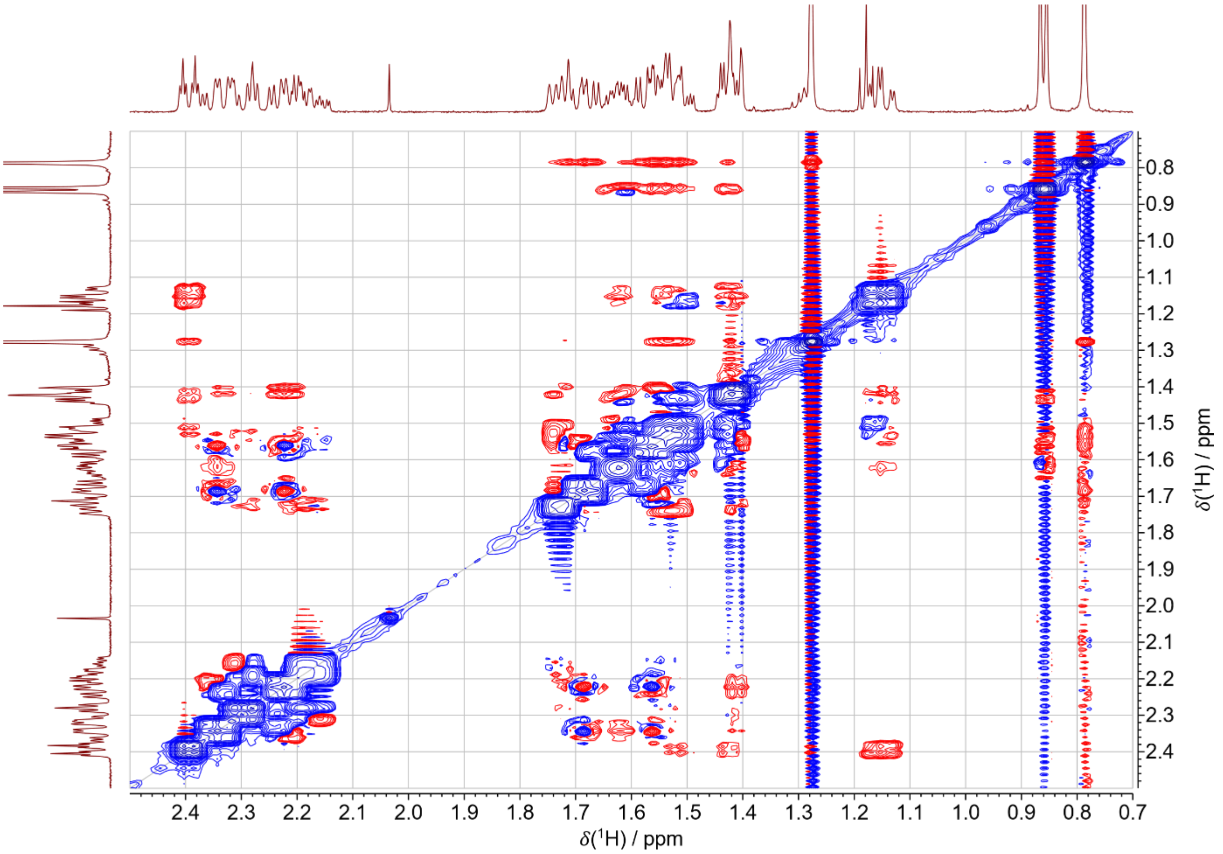


**Figure S14.** $^{1}H$–$^{1}H$ NOESY NMR spectrum of (–)-hardwickiic acid (**1**) ($CD_3OD$, 600 MHz, aliphatic region).

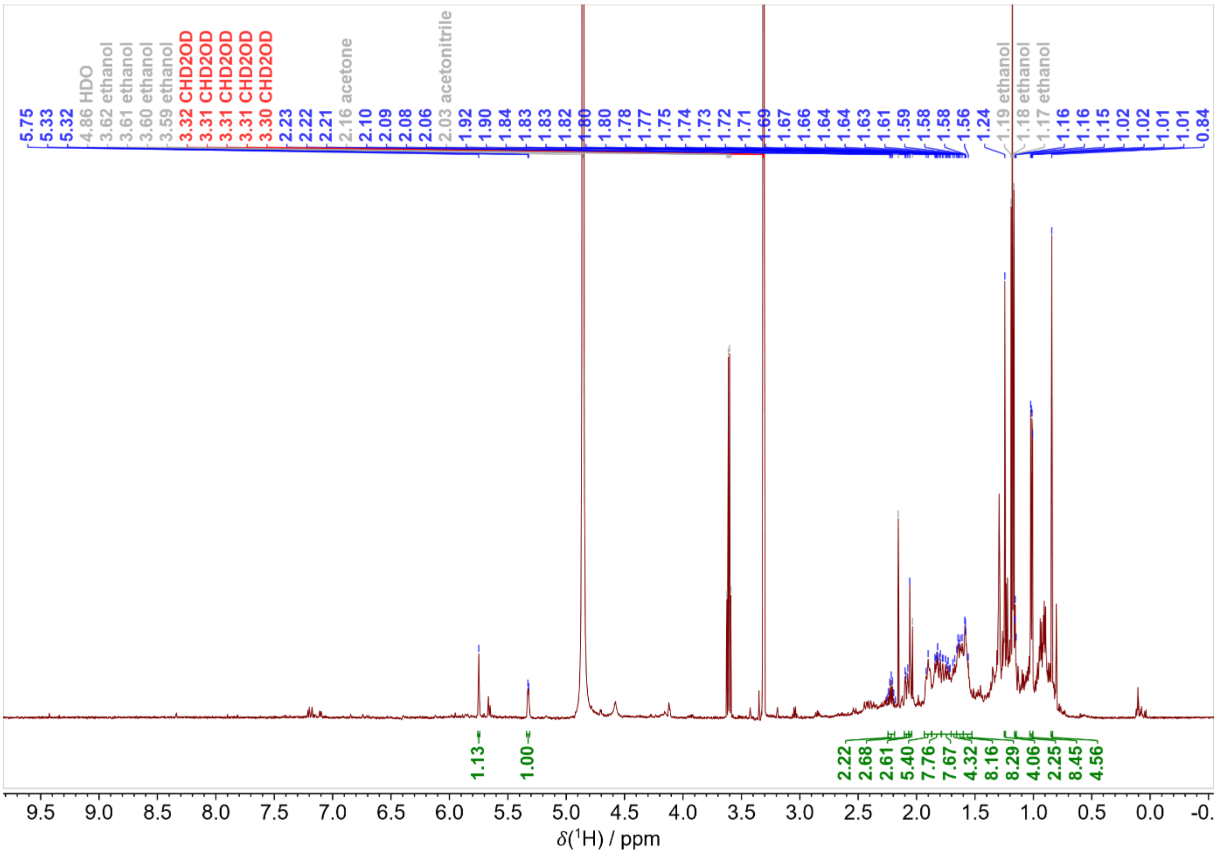


**Figure S15.** $^{1}$H NMR spectrum of (–)-abietic acid (**2**) ($CD_3OD$, 600 MHz).

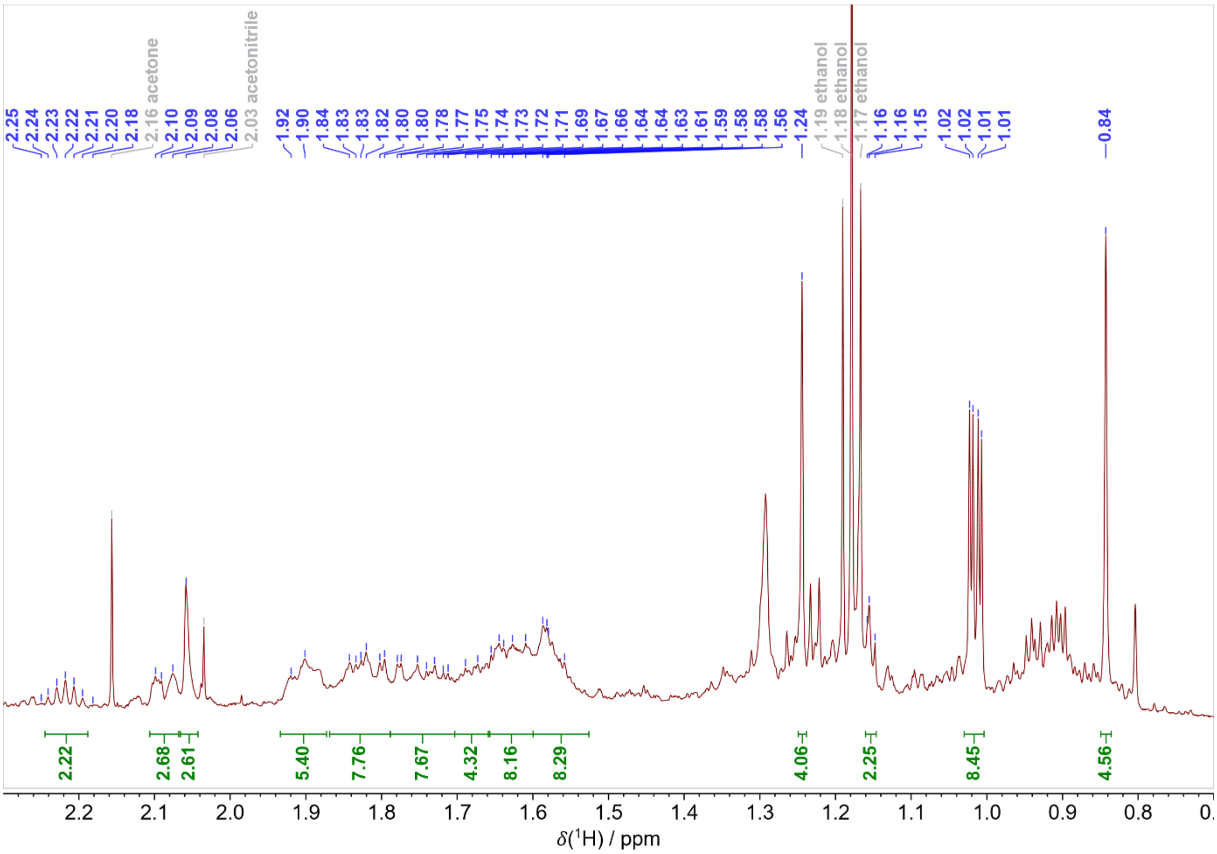

**Figure S16.** $^1$H NMR spectrum of (–)-abietic acid (**2**) ($CD_3OD$, 600 MHz, aliphatic region).

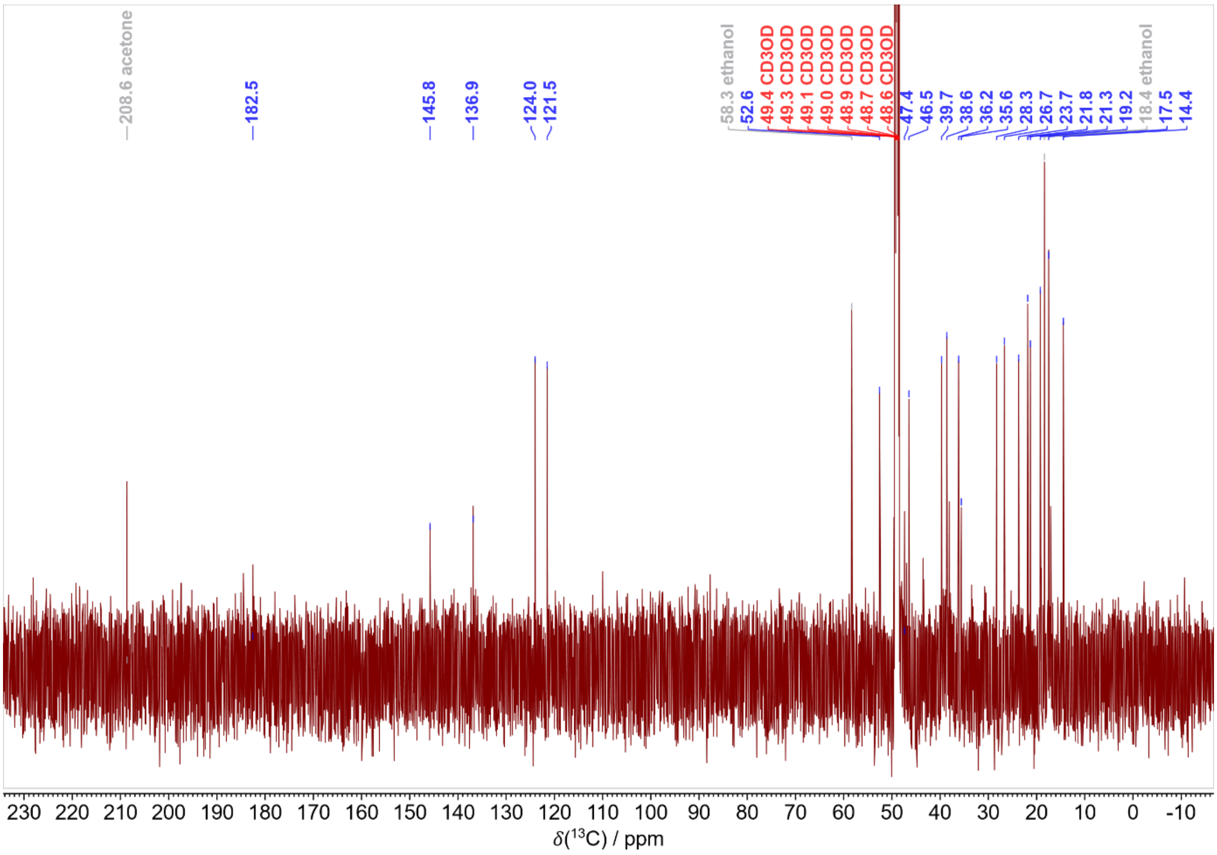


**Figure S17.** $^{13}C$ NMR spectrum of (–)-abietic acid (**2**) ($CD_3OD$, 151 MHz).

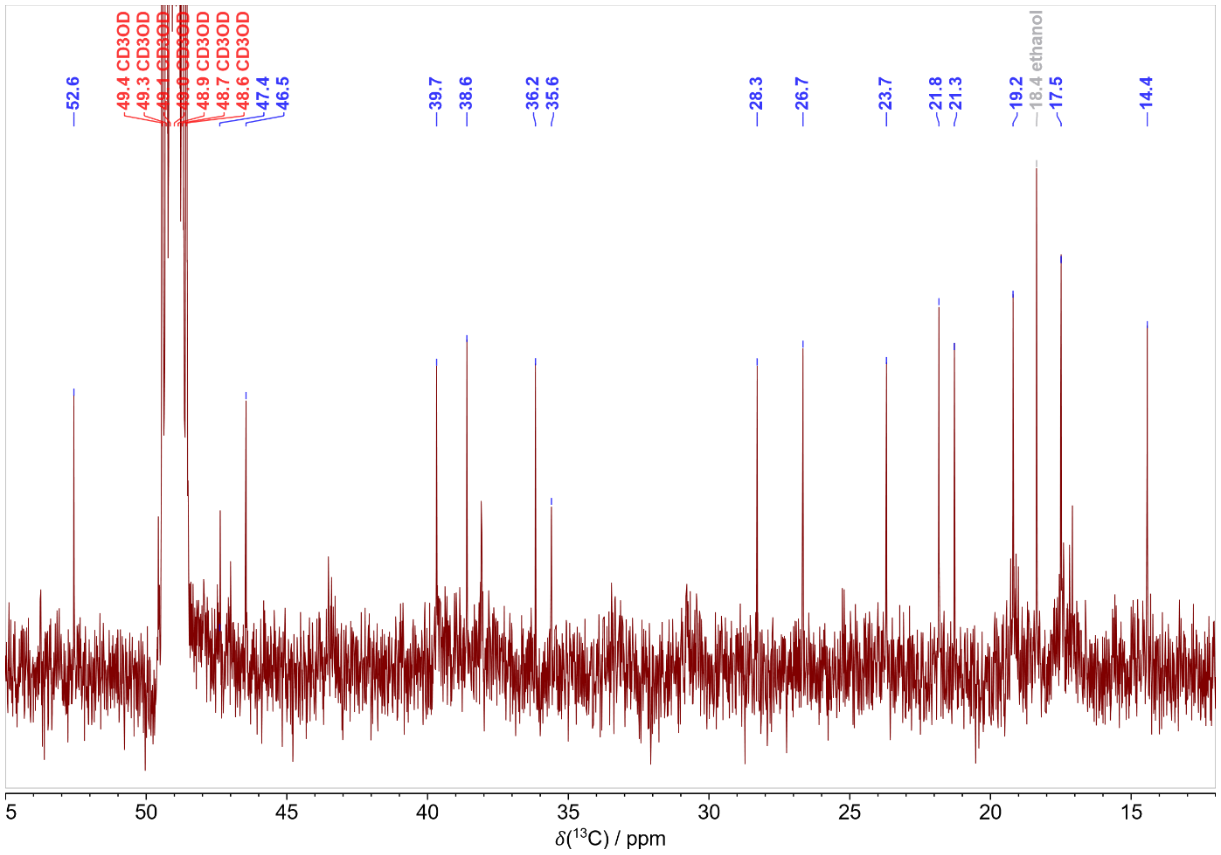


**Figure S18.** $^{13}C$ NMR spectrum of (–)-abietic acid (**2**) ($CD_3OD$, 151 MHz, aliphatic region).

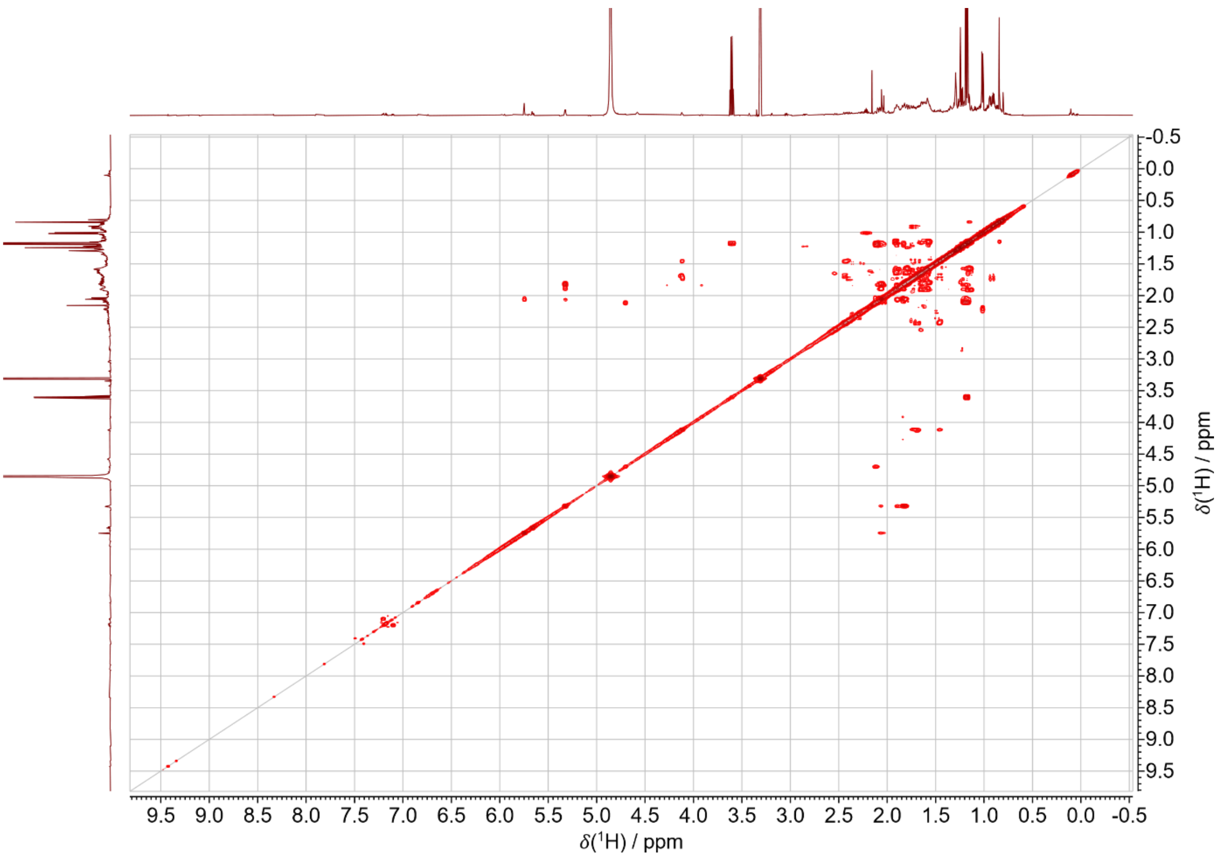


**Figure S19.** $^{1}H$–$^{1}H$ COSY NMR spectrum of (–)-abietic acid (**2**) ($CD_3OD$, 600 MHz).

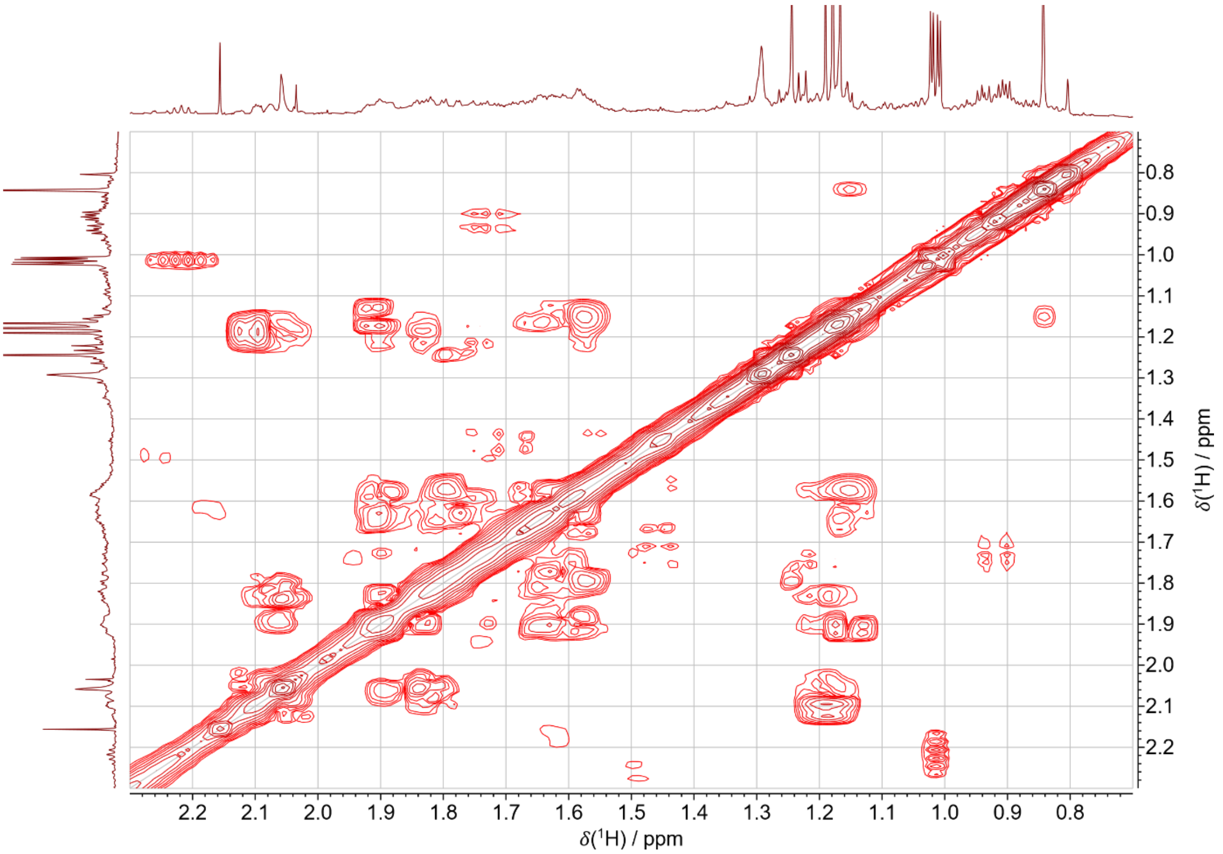


**Figure S20.** ¹H–¹H COSY NMR spectrum of (–)-abietic acid (**2**) ($CD_3OD$, 600 MHz, aliphatic region).

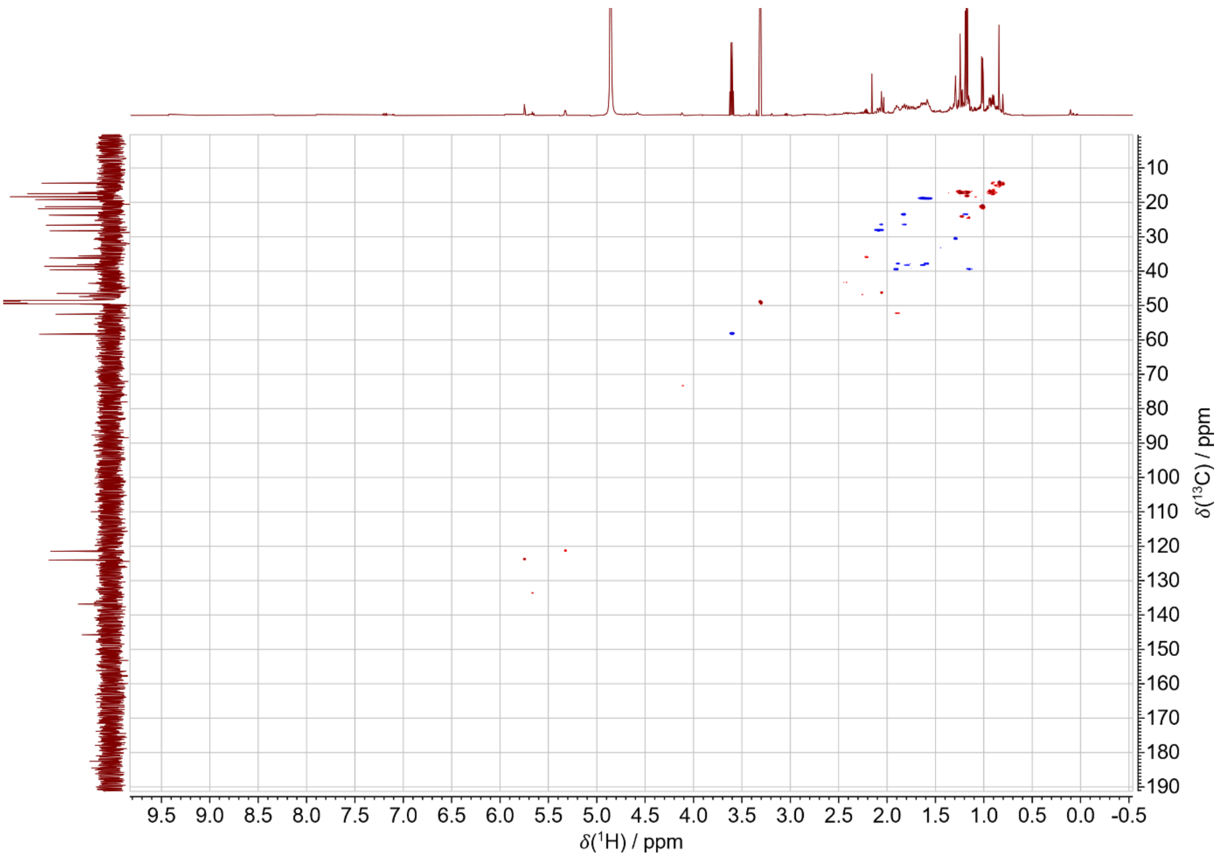


**Figure S21.** $^{1}H$–$^{13}C$ edHSQC NMR spectrum of (–)-abietic acid (**2**) ($CD_3OD$, 600 and 151 MHz).

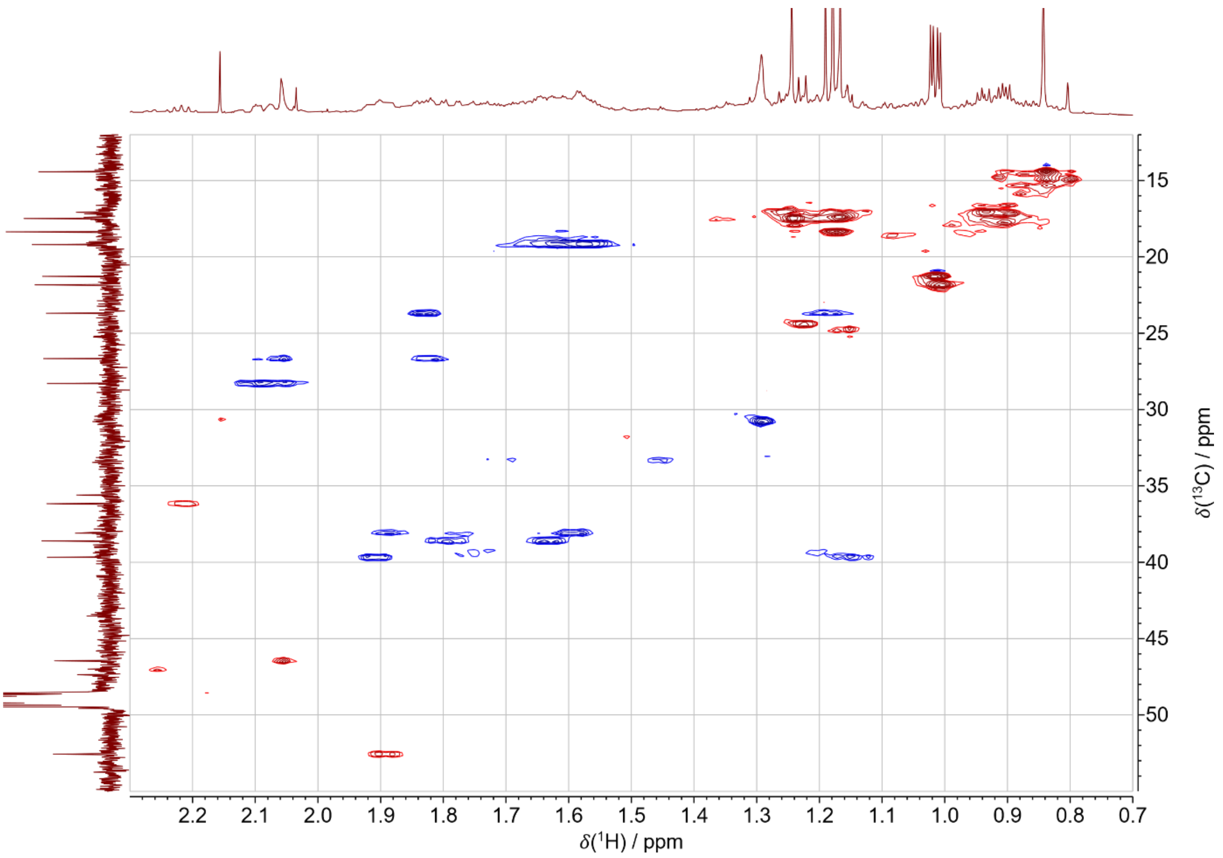


**Figure S22.** $^{1}H$–$^{13}C$ edHSQC NMR spectrum of (–)-abietic acid (**2**) ($CD_3OD$, 600 and 151 MHz, aliphatic region).

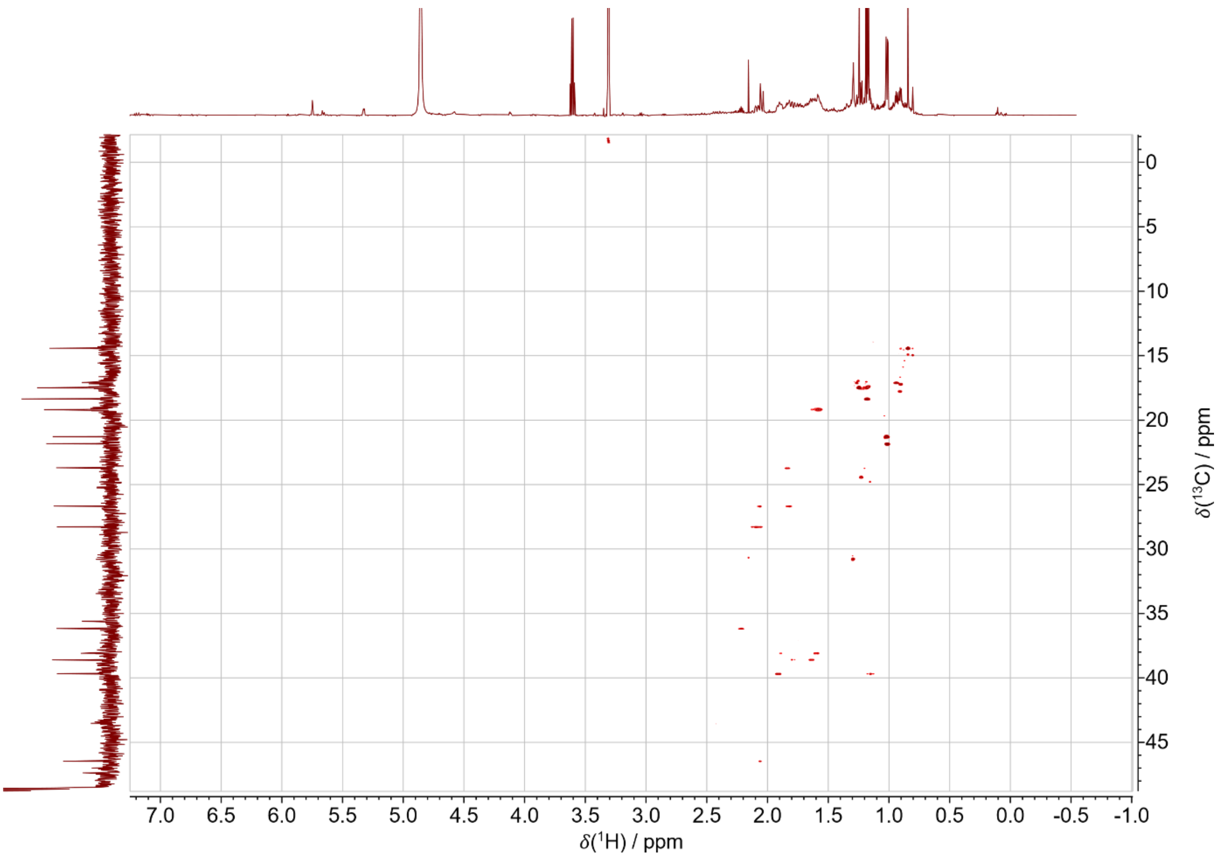


**Figure S23.** $^{1}H$–$^{13}C$ bsHSQC NMR spectrum of (–)-abietic acid (**2**) ($CD_3OD$, 600 and 151 MHz).

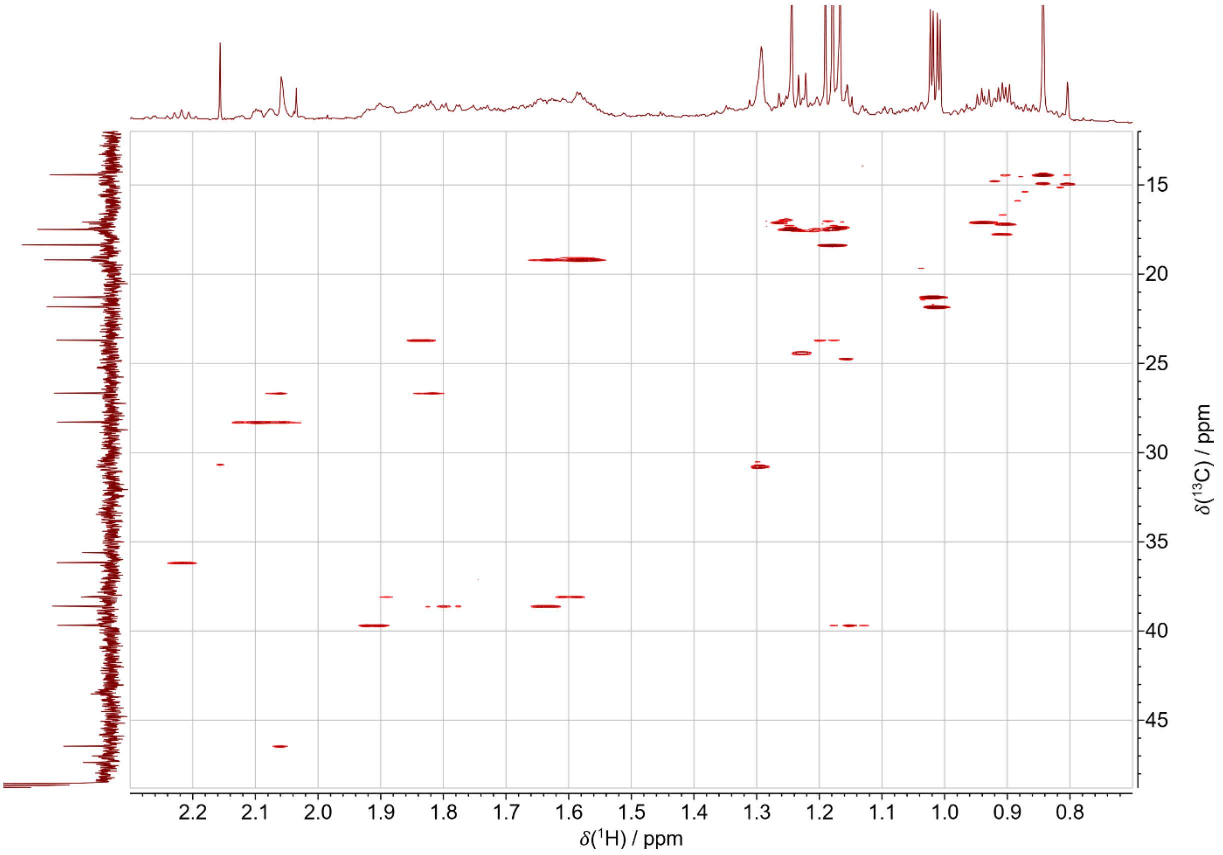


**Figure S24.** $^{1}H$–$^{13}C$ bsHSQC NMR spectrum of (–)-abietic acid (**2**) ($CD_3OD$, 600 and 151 MHz, aliphatic region).

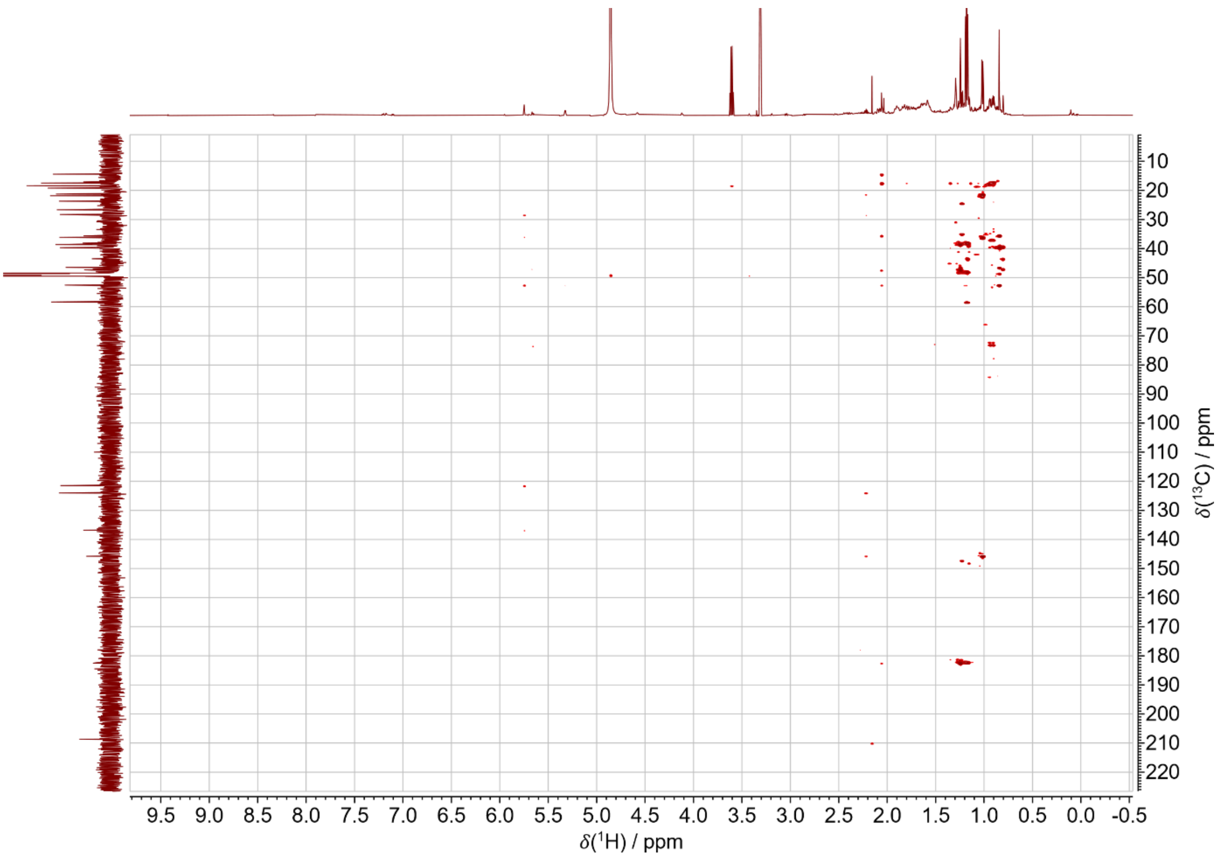


**Figure S25.** $^{1}H$–$^{13}C$ HMBC NMR spectrum of (–)-abietic acid (**2**) ($CD_3OD$, 600 and 151 MHz).

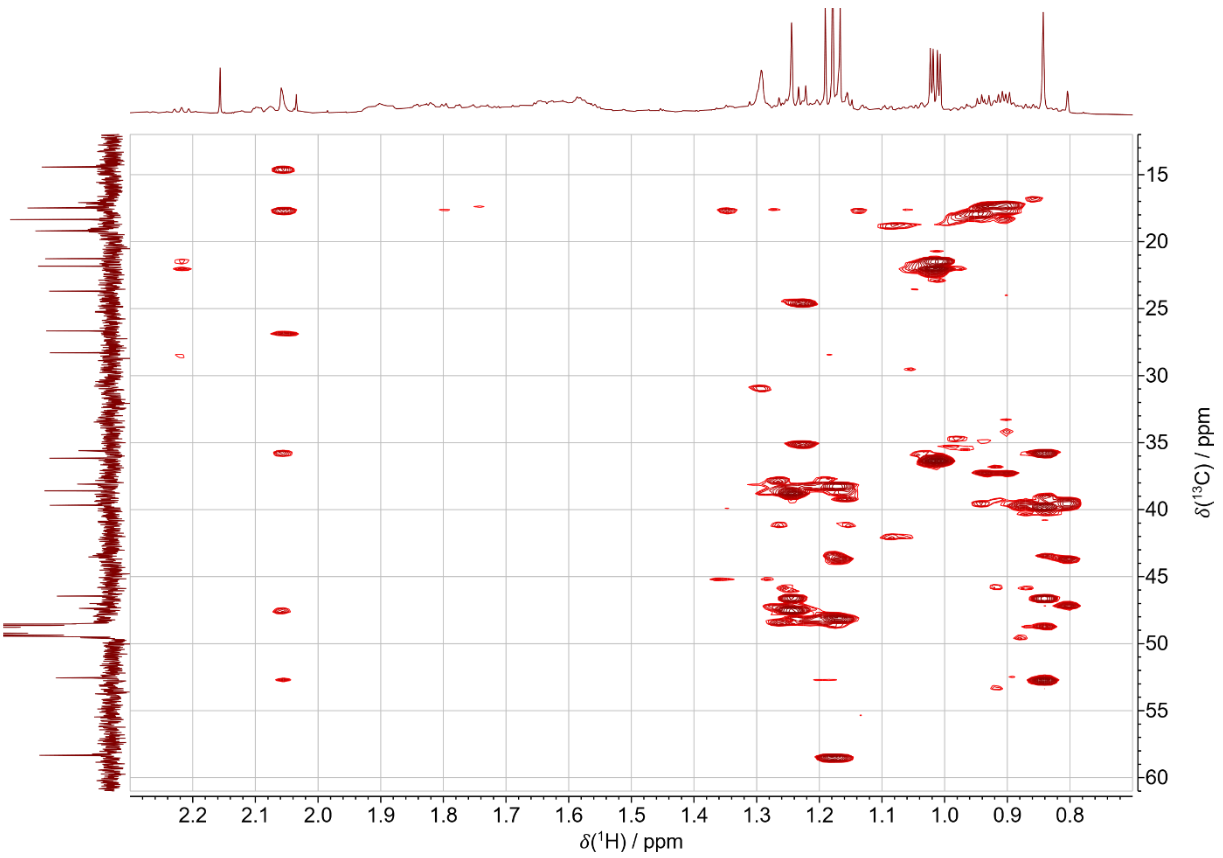


**Figure S26.** $^{1}H$–$^{13}C$ HMBC NMR spectrum of (–)-abietic acid (**2**) ($CD_3OD$, 600 and 151 MHz, aliphatic region).

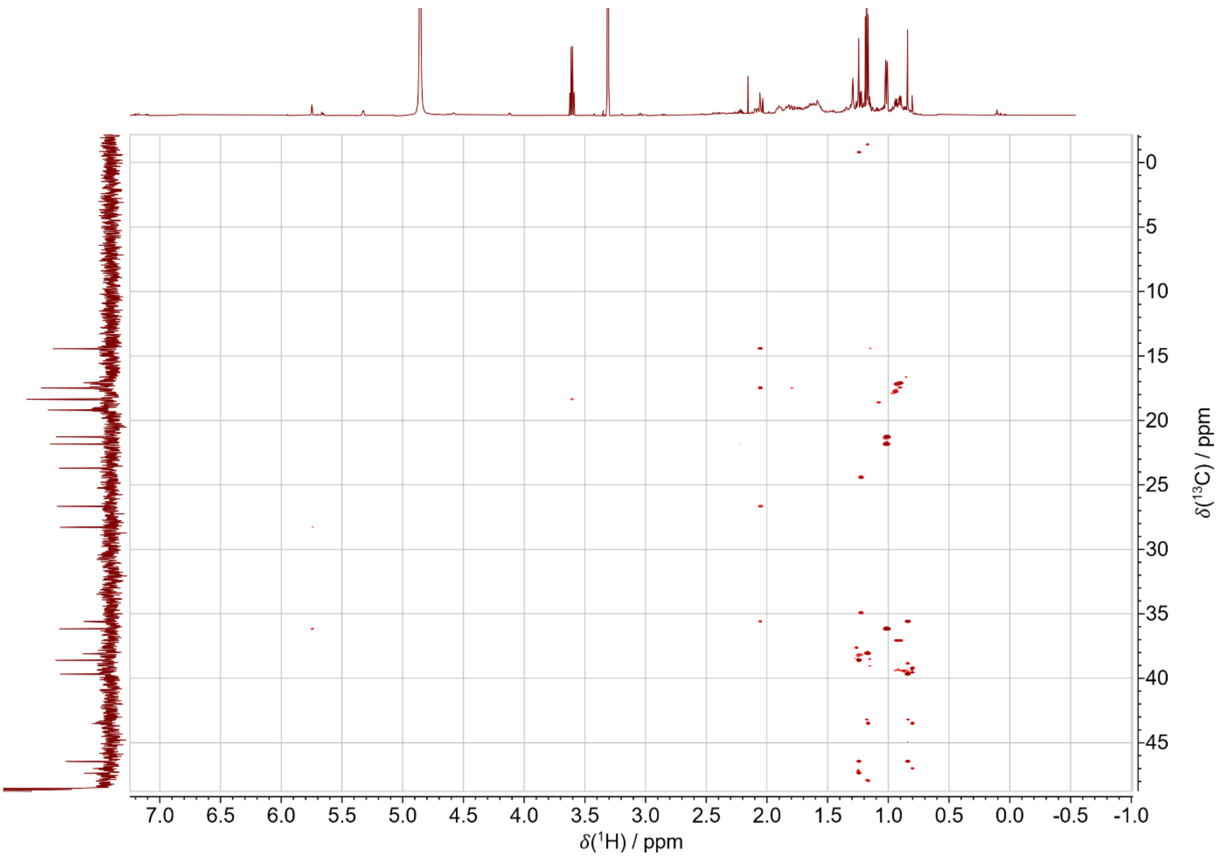


**Figure S27.** $^{1}H$–$^{13}C$ bsHMBC NMR spectrum of (–)-abietic acid (**2**) ($CD_3OD$, 600 and 151 MHz).

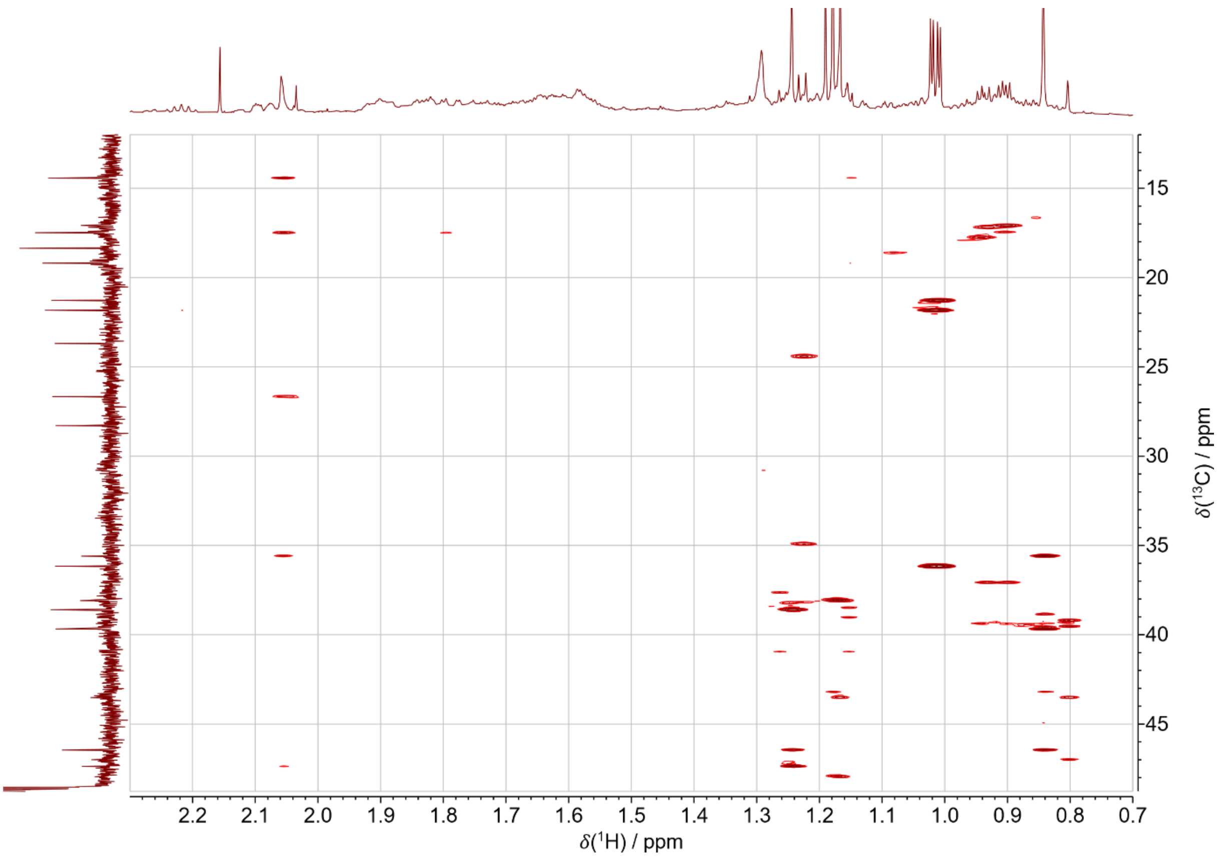


**Figure S28.** $^{1}H$–$^{13}C$ bsHMBC NMR spectrum of (–)-abietic acid (**2**) ($CD_3OD$, 600 and 151 MHz, aliphatic region).

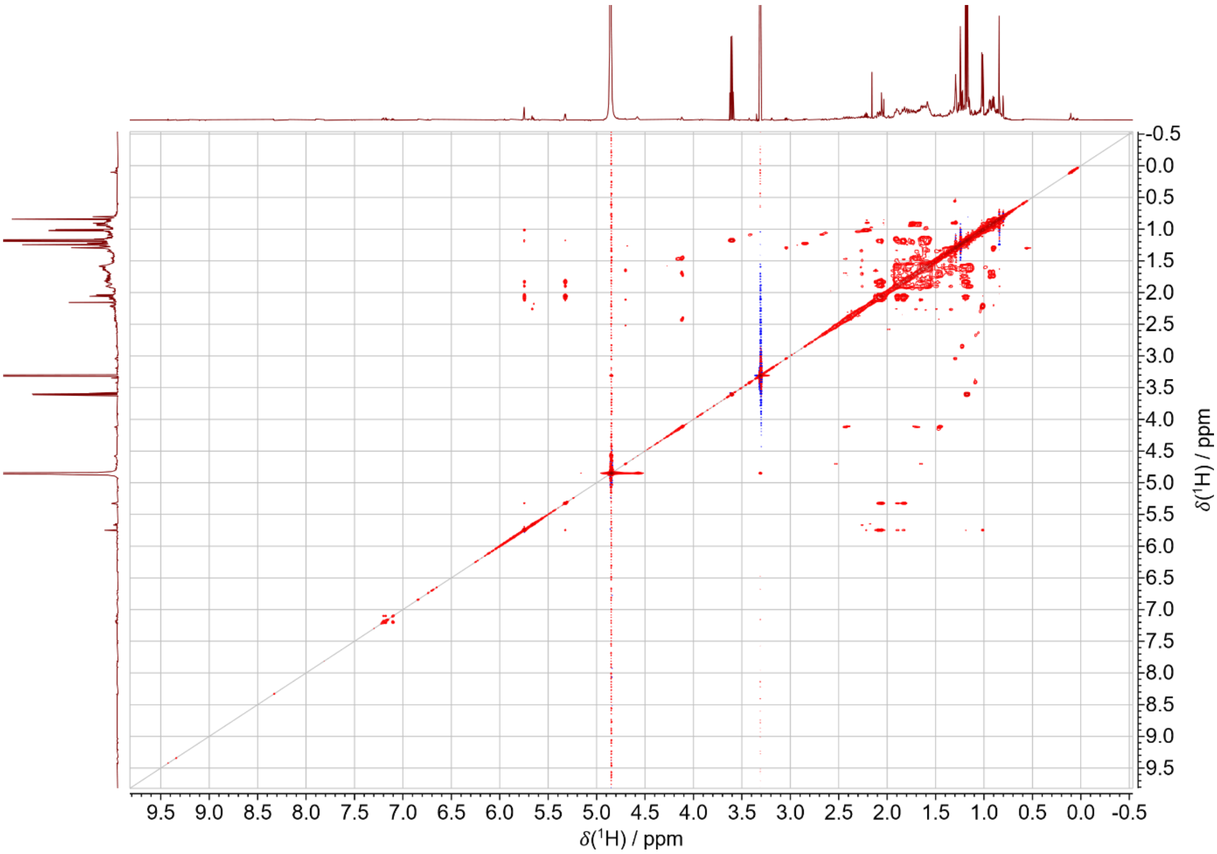


**Figure S29.** $^{1}$H–$^{1}$H TOCSY NMR spectrum of (–)-abietic acid (**2**) ($CD_3OD$, 600 MHz).

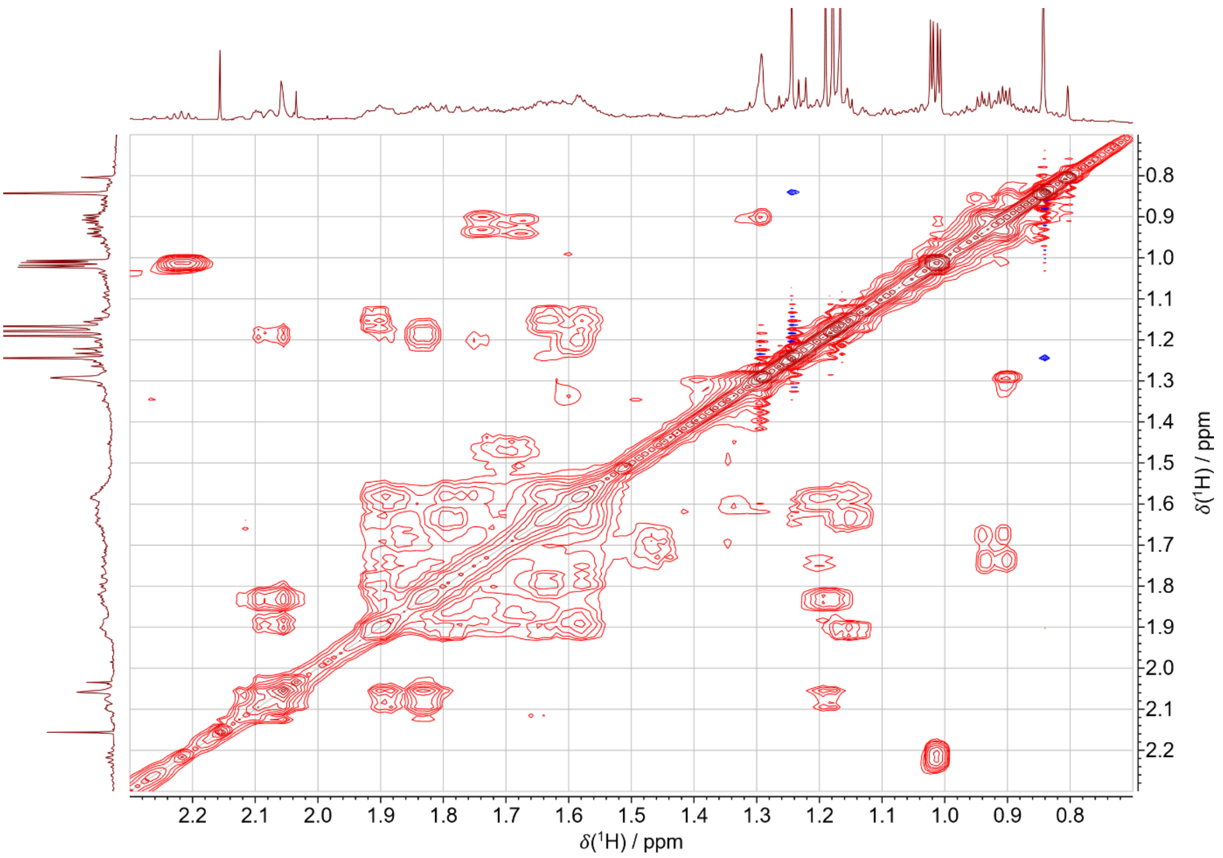


**Figure S30.** $^{1}H$–$^{1}H$ TOCSY NMR spectrum of (–)-abietic acid (**2**) ($CD_3OD$, 600 MHz, aliphatic region).
